# Genomic investigations provide insights into the mechanisms of resilience to heterogeneous habitats of the Indian Ocean in a pelagic fish

**DOI:** 10.1038/s41598-021-00129-5

**Published:** 2021-10-19

**Authors:** Wilson Sebastian, Sandhya Sukumaran, S. Abdul Azeez, K. R. Muraleedharan, P. K. Dinesh Kumar, P. U. Zacharia, A. Gopalakrishnan

**Affiliations:** 1 Marine Biotechnology Division, ICAR-Central Marine Fisheries Research Institute, Ernakulam North P.O., Kochi, Kerala 682018 India; 2 CSIR-National Institute of Oceanography, Regional Centre Kochi, Dr Salim Ali Road, Post Box No. 1913, Kochi, Kerala India

**Keywords:** Ecological genetics, Molecular ecology

## Abstract

The adaptive genetic variation in response to heterogeneous habitats of the Indian Ocean was investigated in the Indian oil sardine using ddRAD sequencing to understand the subpopulation structure, stock complexity, mechanisms of resilience, and vulnerability in the face of climate change. Samples were collected from different ecoregions of the Indian ocean and ddRAD sequencing was carried out. Population genetic analyses revealed that samples from the Gulf of Oman significantly diverged from other Indian Ocean samples. SNP allele-environment correlation revealed the presence of candidate loci correlated with the environmental variables like annual sea surface temperature, chlorophyll-*a*, and dissolved oxygen concentration which might represent genomic regions allegedly diverging as a result of local adaptation. Larval dispersal modelling along the southwest coast of India indicated a high dispersal rate. The two major subpopulations (Gulf of Oman and Indian) need to be managed regionally to ensure the preservation of genetic diversity, which is crucial for climatic resilience.

## Introduction

The dynamics of marine connectivity is complex, operating over large geographical ranges across historical and contemporary periods and affected by environmental and oceanographic factors^1^. In marine fishes, the dispersal of individuals at different life stages, mainly larvae, juveniles, and adults can contribute disproportionally to connectivity and gene flow. Even though marine pelagic fishes are considered to be highly dispersive with significant amounts of gene flow, recent evidence using advanced genomic tools indicates the presence of locally adapted populations with restricted gene flow^2^. Therefore, it is also important to understand the patterns of the partitioning of genetic variation within and among populations^3^ for identifying conservation units and developing fishery management plans, as well as understanding climatic impacts.

Marine fishes are considered to be less genetically structured than freshwater fishes^4^. The low degree of genetic differentiation in marine fishes has been traditionally hypothesized to be the result of the low ecological heterogeneity (compared to freshwater), lack of dispersal barriers, and large effective population size^4^. However, recent investigations challenge these concepts as extensive genomic heterogeneity has been reported in marine fishes employing putative adaptive loci^5,6^ Both dispersal and local adaptation can be considered as strategies for coping with environmental change, and when dispersal rates are high compared to the evolutionary rescue by local adaptation, dispersal can reduce local adaptation. On the contrary, when local adaptation occurs faster than dispersal, evolutionary rescue dominates, causing monopolization effects by a species^7^. Yet, local adaptation and adaptive divergence are possible in spite of high gene flow, when adaptive alleles occur in tightly linked regions of the genome where recombination is not possible^8^.

Partitioning of genetic variation within and among populations has a profound influence on species resilience, and several approaches have been employed to understand these patterns^9^. Traditionally, these studies were limited to few genetic markers (often with insufficient genetic information) creating problems in interpreting the results (especially identifying recent demographical events) and making conclusions^10^.

Next-generation sequencing has enabled genomic approaches to sample large parts of the genome even in non-model organisms^11^. These genome-wide scans can sample loci from the so-called “genomic islands of divergence” or regions with low recombination where adaptive signals can be observed despite the level of gene flow across populations^12^. Using a population genomic approach with restriction site-associated DNA sequencing (RADseq), a method that sequences the DNA flanking specific restriction sites in the genome^13^, the detection of genome-wide variation like single nucleotide polymorphisms (SNPs) can be accomplished by sampling the same genomic region across individuals, thus generating a reduced representation of the genome^14^. The RADseq approach has been applied in many non-model organisms to develop thousands of SNPs^15^, linkage maps^16^, microsatellite markers^17^, undertake genome scans^18^, detect population differentiation^19^, and phylogeography^20^ using various protocols^5,14^. Examples of genomic investigations on fishes include studies on the three-spined stickleback, *Gasterosteus aculeatus*, which provided insights regarding the diversification of populations into three life forms (marine, anadromous and freshwater)^5^, and on cichlids of African lakes^21^ providing information regarding their massive diversification that happened during the past 10 million years^22^. All these studies indicated the presence of adaptive diversity patterns not detected by neutral markers and revealed an additional layer of genetic diversity that needs conservation and management.

Indian oil sardine, *Sardinella longiceps,* is one of the very important pelagic fish of the Indian Ocean and supported the largest pelagic fishery of India with an average annual production of 0.572 million tons during the period 1950–2014^23^. India has been the major contributor of oil sardine among the Indian ocean countries that harvest oil sardine as it contributes up to 80% of the global oil sardine catch (other major harvesters are Oman, Yemen, Iran and Pakistan)^23^. The annual harvest in India was 0.34 million tons in 2017 but a decline in abundance was recorded in 2019 with Indian oil sardine occupying the 9th position in landings (0.15 million tons). Ribbon fishes, lesser sardines and Indian mackerel formed the largest pelagic fishery in 2019, replacing Indian oil sardine^24^. Historically, sardine fishery has exhibited phases of decline followed by abundance in response to fluctuations in environmental parameters^25–27^. Availability of nitrogen by upwelling and other mixing processes, especially runoff from rivers^28^, also affects the productivity of oceanic habitats affecting the distribution and the abundance of sardines. Overfishing along with the alterations in the timing of the southwest monsoon, probably induced by climate change, have been presumed to be other reasons for the fishery decline^29^.

Indian oil sardine is considered a cheap source of protein for millions of people and is relished as a delicacy along the southwest coast of India. Being a forage fish, its importance in trophic ecology and the food web cannot be overemphasized. Sardine dwells in coastal waters with a depth up to 30 m and is characterized by relatively high levels of fecundity and a pelagic larval duration of around 40–50 days^30^. They are distributed widely across tropical latitudes indicating the success of this species in colonizing varied habitats and subsequently making them excellent models for genomic investigations on adaptive evolution and divergence. Despite the possibility of high levels of dispersal, significant genetic structuring and locally adaptive variation have been indicated in sardines in previous studies^31,32^. The present authors have extensively investigated genetic structuring, phenotypic divergence and adaptation patterns using mitochondrial and microsatellite markers^31–34^. Mitochondrial markers revealed a lack of genetic differentiation in Indian oil sardines, whilst microsatellite markers detected significant genetic differentiation^31^. Comparative investigations using complete mitochondrial genomes indicated the presence of positive selection and local adaptation mainly along the southwest Indian Ocean, a region characterized by high variations in sea surface temperature, dissolved oxygen, and chlorophyll-*a*^32^. Phenotypic divergence has also been reported in Indian oil sardine indicating adaptive variation^34^. Wide variations in temperature, salinity, dissolved oxygen, and chlorophyll-*a* have been reported throughout the distribution of Indian oil sardine across the Indian Ocean^32^. These physical and biological barriers to dispersal, along with mismatches between phenotype and environment in addition to energetic costs of larval dispersal, could induce mortality of maladapted phenotypes and reduce connectivity substantially in the oceanic environment^35^.

Understanding genome-wide patterns of genetic diversity are crucial to devising conservation and management measures for this important species in Indian waters. Management measures should consider stock complexity which otherwise may lead to erosion of spawning components and depletion of the over-exploited populations^36^. Fishery management in Indian waters occurs mainly through seasonal closures and mesh size regulations^37,38^, and species-specific management plans have not been implemented for the Indian oil sardine fishery despite the decline in landings. Additionally, knowledge regarding genomic patterns of divergence is crucial to understand and predict the response of Indian oil sardines to habitat variability and climate change in the Indian Ocean. We explored the spatial patterns of adaptive variation and genetic differentiation among sardine populations collected from the Northern Arabian Sea, southeast Arabian Sea, and Bay of Bengal by employing genome-wide genetic markers derived from ddRAD sequencing. The reduced representation genomic data was further analyzed to detect candidate SNP loci that may be indicative of local adaptation and loci associated with environmental gradients. We also investigated the larval transport patterns using a validated hydrodynamic model coupled with a particle tracking module along the southwest coast of India during summer and winter monsoons.

## Results

### Genotyping and SNP discovery

All the sequenced samples passed the criteria of a minimum number of raw reads of < 1,000,000. After de novo processing using the 'denovo_map.pl' program in STACKS, 50,076 polymorphic RAD loci with 1SNP and two alleles were retained. Finally, 49,361 polymorphic loci conformed to Hardy Weinberg Equilibrium and read depth of < 10 per stack, representing 100 samples from five populations selected for population genomic analyses (Supplementary Tables S1 and S2).

### Genome-wide genetic diversity

The average frequency of major alleles (P) and average observed heterozygosity (Ob Het) of both fixed and variable sites from five populations ranged from 0.998 to 0.999 and 0.0017–0.0020, respectively. Whereas for the variable sites, the *p*-value decreased to 0.85–0.82 and Ob Het increased to 0.178–0.198. The overall nucleotide diversity (π) in *S. longiceps* populations ranged from 0.0015 to 0.0028 and samples from the Oman Sea—OMAN (0.0020) and North Eastern Arabian Sea—NEAS (0.0015) had the lowest level of nucleotide diversity. The population of South-Eastern Arabian Sea—SEAS (0.0026), Southern Bay of Bengal—SBoB (0.0025), and Northern Bay of Bengal—NBoB (0.0028) had relatively high diversity when compared to OMAN and NEAS. All the five populations had unique (private) sites/alleles with the maximum in SEAS (970) and minimum in NEAS (387) (Table 1).

**Table 1:** Summary of Genetic diversity statistics for restriction-site associated DNA (RAD) sites of *S. longiceps*.

# Pop ID	Private	Sites	Variant sites	Polymorphic sites	% Polymorphic loci	NumIndv	Var	P	Obs Het	Obs Hom	Exp Het	Exp Hom	π	F_IS_
**# All positions (variant and fixed)**														
OMAN	387	4,393,352	44,876	25,709	0.5852	12.6394	0.4642	0.9984	0.002	0.998	0.0022	0.9978	0.0020	0.0008
NEAS	88	4,256,641	27,963	20,907	0.4912	11.4331	2.1912	0.9991	0.0012	0.9988	0.0014	0.9986	0.0015	0.0007
SEAS	970	4,691,531	46,269	44,683	0.9524	23.1396	11.4238	0.9983	0.0018	0.9982	0.0025	0.9975	0.0026	0.0025
SBoB	544	4,682,365	45,620	39,288	0.8391	12.0171	3.9287	0.9984	0.0017	0.9983	0.0024	0.9976	0.0025	0.0022
NBoB	563	4,721,272	49,088	42,020	0.89	11.848	1.8672	0.9982	0.002	0.998	0.0026	0.9974	0.0028	0.0021
**# Variant positions**														
OMAN	387	–	–	–	–	12.3226	0.6209	0.8425	0.198	0.802	0.211	0.789	0.2398	0.0818
NEAS	88	–	–	–	–	11.4249	1.7164	0.8555	0.1821	0.8179	0.2093	0.7907	0.225	0.1139
SEAS	970	–	–	–	–	20.0284	13.4432	0.8255	0.1851	0.8149	0.2534	0.7466	0.2601	0.2583
SBoB	544	–	–	–	–	10.2993	4.8389	0.8308	0.1781	0.8219	0.2429	0.7571	0.256	0.2246
NBoB	563	–	–	–	–	10.015	2.4759	0.8224	0.1954	0.8046	0.2524	0.7476	0.2678	0.1985

Population ID (Pop ID), the number of variable sites unique to each population (Private), the number of nucleotide sites across the data set (Sites), polymorphic sites across the data set (Polymorphic Sites), percentage of polymorphic loci (% polymorphic loci), the average number of individuals genotyped at each locus (NumIndv), variance (Var), the average frequency of the major allele (P), the average observed heterozygosity per locus (Obs Het), the average observed homozygosity (Obs Hom), the average expected heterozygosity (Exp Het), the average expected homozygosity (Exp Hom), the average nucleotide diversity (π) and Average Wright’s inbreeding coefficient (F_IS_).

In all the populations, the inbreeding coefficients (F_IS_) of all restriction-site associated DNA (RAD sites) (variant and fixed) ranged from 0.0007 to 0.0025 but these were higher for variant sites only (0.0818 to − 0.2583). The allele frequency spectrum of major alleles across the loci was skewed towards 1.00 and varied slightly across the populations (Supplementary Fig. S1). The highest percentage of allele frequency was observed in category one (rightmost category equal to 1.0 in the graph), indicating that most polymorphic loci across the population are fixed within each population. A lower range of allele frequencies (0.5–0.99) was observed in all the populations as expected for an older genetically diverse population at or near evolutionary equilibrium^39^.

### Genome-wide genetic differentiation

Oman population was highly differentiated from all the other populations (F_ST_/R_ST_ value of 0.0789/0.07632, 0.0657/0.06627, 0.06979/0.06958 and 0.06791/0.06791 for the four pairwise comparisons), in comparisons of pairwise genetic differentiation (F_ST_ and R_ST_) (Table 2, Supplementary Fig. S2) with highly significant *p*-values (< 0.001). In all the other pairwise comparisons, the highest genetic differentiation was observed between the NEAS and NBoB followed by the SEAS and SBoB populations, but *p*-values were not significant. The Oman population was also highly separated along PC1 in PCA plots as compared to other populations while the majority of the individuals of NEAS were separated from others along PC2 (Supplementary Fig. S3). Individuals from SEAS, NBoB and SBoB formed a cluster. The Neighbour-joining tree of populations based on average F_ST_ values of SNPs loci also corroborated these findings (Supplementary Fig. S4).

**Table 2:** Pairwise comparison of genetic divergence (F_ST_, R_ST_) among *S. longiceps* populations.

Populations ID	OMAN	NEAS	SEAS	SBoB	NBoB
OMAN	0	0.001**	0.001**	0.001**	0.001**
NEAS	0.07589, 0.07632	0	0.152	0.230	0.396
SEAS	0.0657, 0.06627	0.0009, 0.001	0	0.118	0.121
SBoB	0.06979, 0.06958	0.00074, 0.00063	0.0001, 0.0003	0	0.396
NBoB	0.06791, 0.06791	0.00159, 0.00132	0.00087, 0.00074	0.00094, 0.00047	0

Below diagonal; genetic divergence among populations as measured by F_ST_, R_ST_. Above diagonal; *p*-value of exact G test for each population pair across all loci by Fisher's method.

*OMAN* Oman Sea, *NEAS* North East Arabian Sea, *SEAS* South East Arabian Sea, *SBoB* South West Bay of Bengal, *NBoB* Northwest Bay of Bengal.

**Highly significant*.*

**Figure 1:**
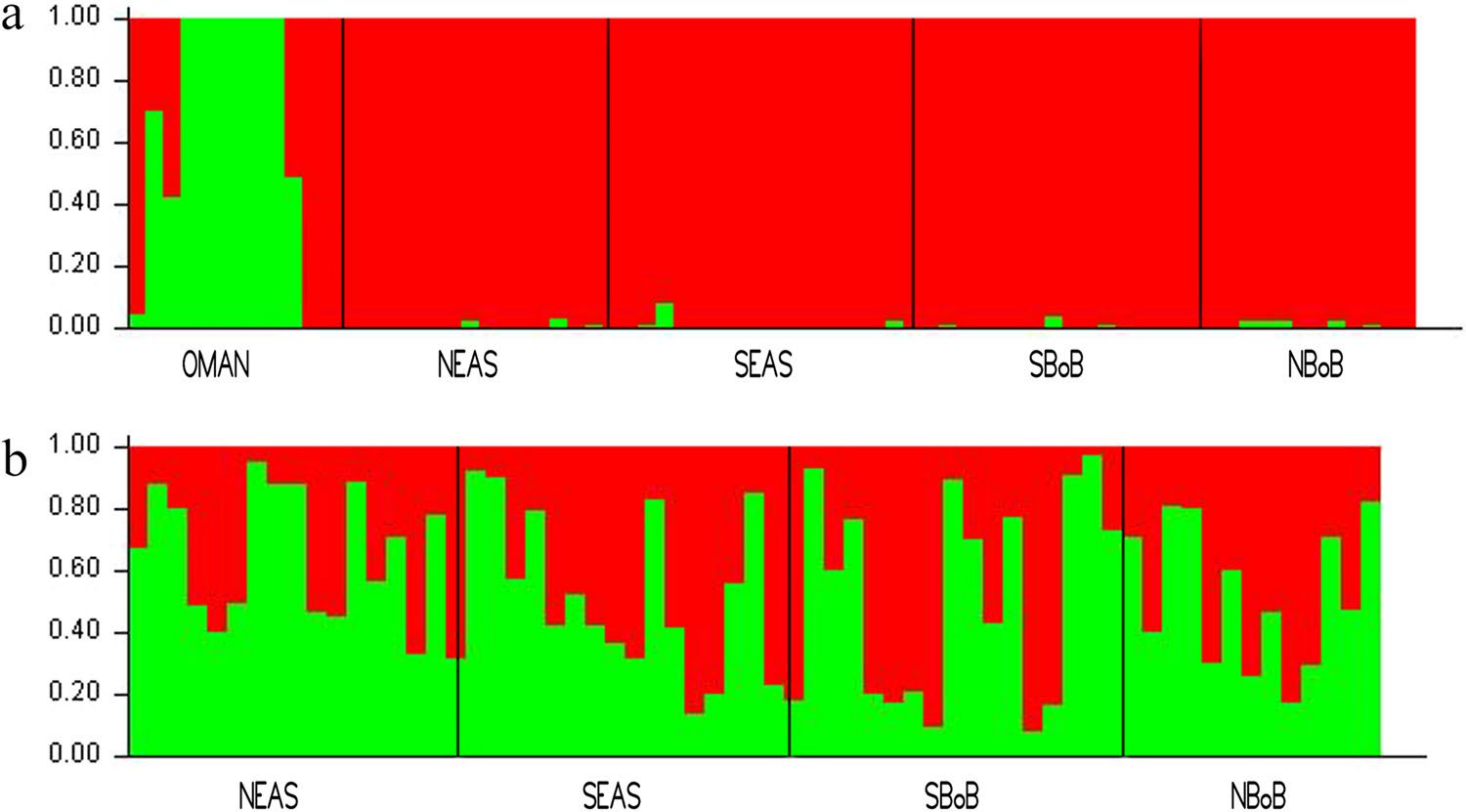
Graphical results of admixture analysis among all populations derived from 56,358 SNPs loci in STRUCTURE. Vertical lines represent the probability of individual membership in simulated clusters. (**a**) Plot for K = 2 (including all the five samples), (**b**) plot for K = 2 (excluding OMAN samples). The plot was generated with STRUCTURE v2.3^85^.

The STRUCTURE analyses using the Bayesian approach also confirmed the result of previous analyses (Fig. 1). The Delta K method^40^ in structure analysis indicated that K = 2 is the best fit model for the data and the plot of posterior probability confirmed these two groups (Supplementary Fig. S17). The second level of analysis, omitting Oman Sea (OMAN) samples also indicated two genetic clusters with varying degrees of representation in each population (Fig. 1). The regression analysis and Mantel test indicated that the populations do not exhibit a pattern of isolation by distance (R square = 0.1681, p 0.2392, mantel test r = 0.182, z = 567 and, p 0.32).

**Figure 2:**
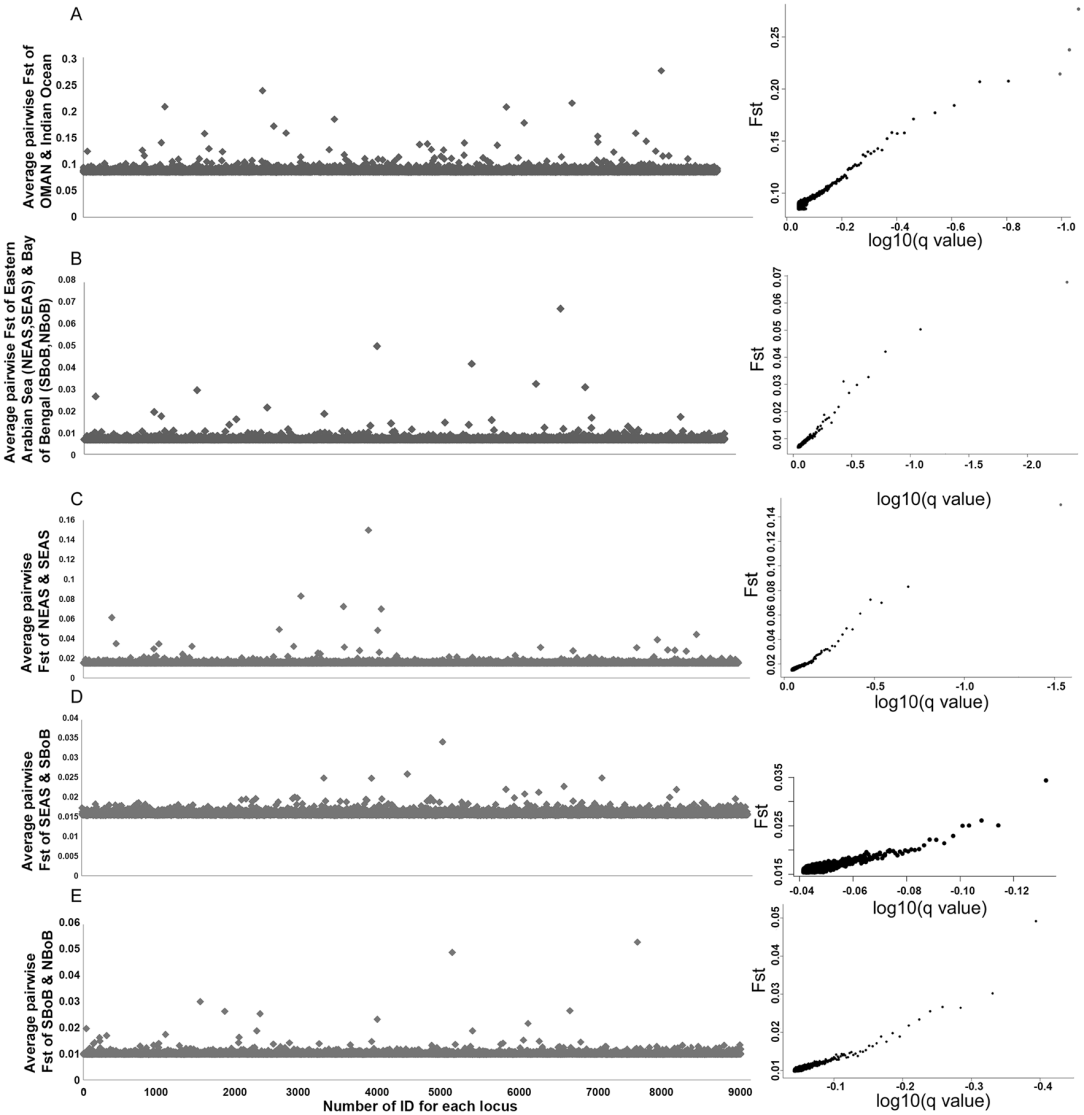
The plot of pairwise F_ST_ of SNP loci potentially subjected to differential selection in *S. longiceps* populations. Pairwise F_ST_ of SNP loci between (**A**) OMAN and the Indian Ocean, (**B**) Arabian Sea (NEAS, SEAS) and Bay of Bengal (SBoB, NBoB), (**C**) NEAS and SEAS, (**D**) SEAS and SBoB, and (**E**) SBoB and NBoB. For the figures on the left, X-axis indicates the number of IDs for each locus and the Y-axis indicates the pairwise F_ST_ values. For the figure on the right, X-axis indicates the false discovery rate (FDR) and Y-axis indicates the pairwise F_ST_ values. The SNP loci with a false discovery rate (FDR) < 0.05 were highlighted in red colour. The plot was generated with R statical package (https://www.r-project.org/).

Detection of genome-wide outlier SNPs across all the populations using BAYESCAN identified five outlier SNPs with F_ST_ values ranging from 0.18 to 0.27. The pairwise comparative analysis between Oman and other Indian Ocean samples identified a total of three outlier SNPs with the highest F_ST_ of 0.27. Only one outlier SNP could be identified to differentiate the eastern Indian Ocean (NBoB and SBoB) and western Indian Ocean (SEAS and NEAS) and no outlier SNPs could be identified for differentiating NBoB, SBoB and SEAS (Fig. 2). The result indicated that the OMAN population was highly diverged genetically as compared to all the other samples. Intermediate and low level of genetic divergence was observed between the eastern Indian Ocean and the western Indian Ocean samples. The positive and negative alpha values of the SNP locus indicated the posterior probability of the effect of directional selection and purifying selection respectively. All the outlier loci have a positive alpha coefficient, indicating that these loci are under positive directional selection (Supplementary Table S6).

### Oceanographic and environmental features and Indian oil sardine fishery

**Figure 3:**
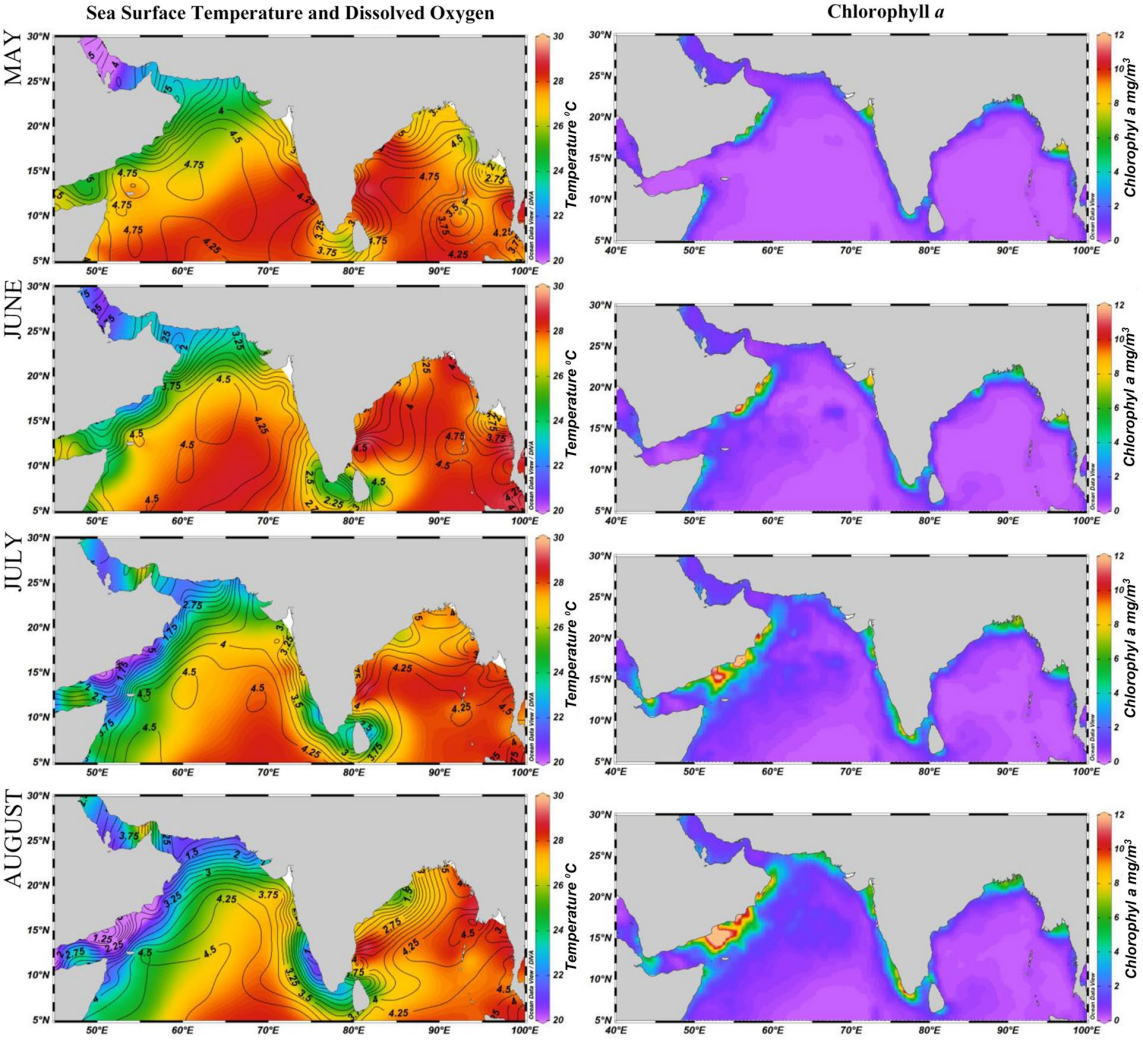
Monthly Chlorophyll-*a* (mg/m^3^), sea surface temperature (°C) and dissolved oxygen (µmol/kg) for the Bay of Bengal and the Arabian Sea from May to August. Chlorophyll-*a* and sea surface temperature gradients are represented as coloured shades. Dissolved oxygen is represented as contour lines. The images were generated in ODV 5.1.7 (https://odv.awi.de/).

Long term trends in the monthly chlorophyll-*a* (Chl-*a*) and sea surface temperature (SST) indicated substantial variations along the coastal regions of OMAN, NEAS, SEAS, SBoB and NBoB. OMAN exhibited the lowest mean SST (25.4 °C) with the highest (24–27 °C) during the northeast monsoon season (October–March) and lowest (20–23 °C) during the southwest monsoon season (June–September) (Supplementary Figs. S5 and S6). In contrast, maximum mean Chl-*a* concentration was noticed in this region with the highest (4–10 mg/m^3^) during the southwest monsoon and lowest (1–3 mg/m^3^) during northeast monsoon season (Supplementary Figs. S5 and S7). The average sea surface salinity (SSS) was also higher in the OMAN throughout the year (36–38 ppt)^41^ (Fig. 3, Supplementary Fig. S5). SEAS exhibits a distinctive bimodal pattern of SST with warming (29–30 °C) during both inter-monsoon seasons (April–May and October–November) and cooling (26–28 °C) during the southwest and northeast monsoon seasons (Fig. 3, Supplementary Figs. S5 and S6). High chlorophyll-*a* concentration was observed along the coastal region between 8° and 14° N, from May to September, peaking during July and August (5–10 mg/m^3^). By October, it recedes to a low (1–2 mg/m^3^) Chl-*a* concentration and continues up to May (Fig. 3, Supplementary Figs. S5 and S7). NEAS undergoes winter cooling during (October-December) northeast monsoon with an average SST of 28 °C (Supplementary Figs. S5 and S6). Maximum chlorophyll-*a* concentration was observed along the coastal region (19°–25°N) during this period with the highest concentration in October and November (6–7 mg/m^3^) (Supplementary Figs. S5 and S7).

Contrary to the Arabian Sea, the Bay of Bengal (SBoB and NBoB) is characterized by the lowest SST (24–26 °C) during November–April and the highest (27–30 °C) during May–October. The temperature in NBoB varied between 25 and 31 °C during November–April and May–October respectively (Supplementary Figs. S5 and S6). Whilst in SBoB, the temperature varied between 27 and 30 °C during November–April and May–October respectively (Supplementary Figs. S5 and S6). Besides, low Chl-*a* (0–3 mg/m^3^) and low SSS (28–33 ppt) were also observed throughout the year (Fig. 3, Supplementary Figs. S5 and S7).

Among these five eco-regions, OMAN associated with the Gulf of Oman upwelling zone (with an average of 195,450.00 tons/year) followed by SEAS associated with Malabar upwelling zone (with an average of 156,910.95 tons/year, 1985–2019) contributed the largest share of Indian oil sardine fishery. Landings of Indian oil sardine exhibited large interannual variability with abundant (above average) and deficient years (below average) during the past 35 years (Supplementary Fig. S8). The highest landing in India was observed during 2011–2012 (with an average of 216,172.6 tons/year) and intermediate during 2002–2007 (with an average of 98,148.32 tons/year). The lowest landing was observed during 1984–1987 (with an average of 24,906.91 tons/year), 1993–1995 (with an average of 16,569.33 tons/year) and 2017–2019 (with an average of 56,099.66 tons/year).

Chlorophyll-*a* has been used as a proxy for productivity and sardine abundance as *S. longiceps* is considered a plankton feeder with a preference for diatoms like *Fragilaria oceanica*^42,43^. It is observed that the average monthly Chl-*a* was low during May to September for years with lower sardine fishery and high for years with higher sardine fishery (Supplementary Fig. S9), which also has been established by previous investigations^29,44^. The temperature of SEAS was above average through May (pre-spawning) to September (spawning and larval development) during years with low sardine fishery, whereas it is below average during years with abundant fishery (Supplementary Fig. S10). This period also coincides with the spawning and larval development of Indian oil sardine and food availability becomes a crucial factor for the successful survival and persistence of larvae.

Circulations in the Indian Ocean change their direction in the semi-annual scale due to the semi-annual reversal of monsoon over the Indian subcontinent. The major surface currents in the Indian Ocean are summarised in Supplementary Fig. S11. During the winter (northeast monsoon), the surface current systems in the Indian Ocean are similar to the general circulation patterns in the Pacific and Atlantic oceans. The Equatorial Counter Current (ECC), the Northeast Monsoon Current (NMC) and the south equatorial current (SEC, not shown in the Figure) are the major currents observed in this season. Whilst, during the summer (southwest monsoon), the surface currents change remarkably from the other oceans. The eastward flowing Southwest Monsoon Current (SMC), replaces the westward flowing NMC and the northward-flowing Somali Current (SC), replaces the southward flowing SC along the Somali coast. A unique surface flow known as Equatorial Jet (EJ) is observed during the transition period of the monsoon (April–May and November–December)^45^. Similar to the above open ocean currents, the boundary currents along the coastal region is also undergoing seasonal reversals^46^. The West India Coastal Current (WICC) flows towards the North Pole during winter and towards the South Pole during summer along the west coast of India. While East India Coastal Current (EICC) flows towards the North Pole during winter and towards the South Pole during summer^45^.

One of the unique characteristics of the Oman Sea is the presence of strong coastal upwelling at the western ocean boundary induced by the Somali Current and Findlater jet. The Somali Current is a cold ocean boundary current that runs along the coast of Somalia and Oman and the Findlater jet is a narrow, atmospheric jet that blows diagonally across the Indian Ocean. This flow deflection creates open ocean upwelling and downwelling in its left and right side of the path, resulting in one of the most productive ecosystems in the ocean along the Somali Oman coast^47^. An anti-cyclonic eddy, the Great Whirl generated by the Somali current and the Socotra Gyre can be observed in the Oman Sea during the summer season. The horizontal diameter of the Great Whirl is 400–600 km and the surface current velocity is 1.5–2.0 m/s. Similar to the intense coastal upwelling along the Somali-Oman coast, an intense upwelling is also noticed along the southwest coast of India (Malabar upwelling zone), during the southwest monsoon and becomes the second most productive region in the Indian ocean. But narrow continental shelf and immense freshwater influx overwhelm coastal upwelling in the Bay of Bengal region.

### Loci associated with environmental variables

**Figure 4:**
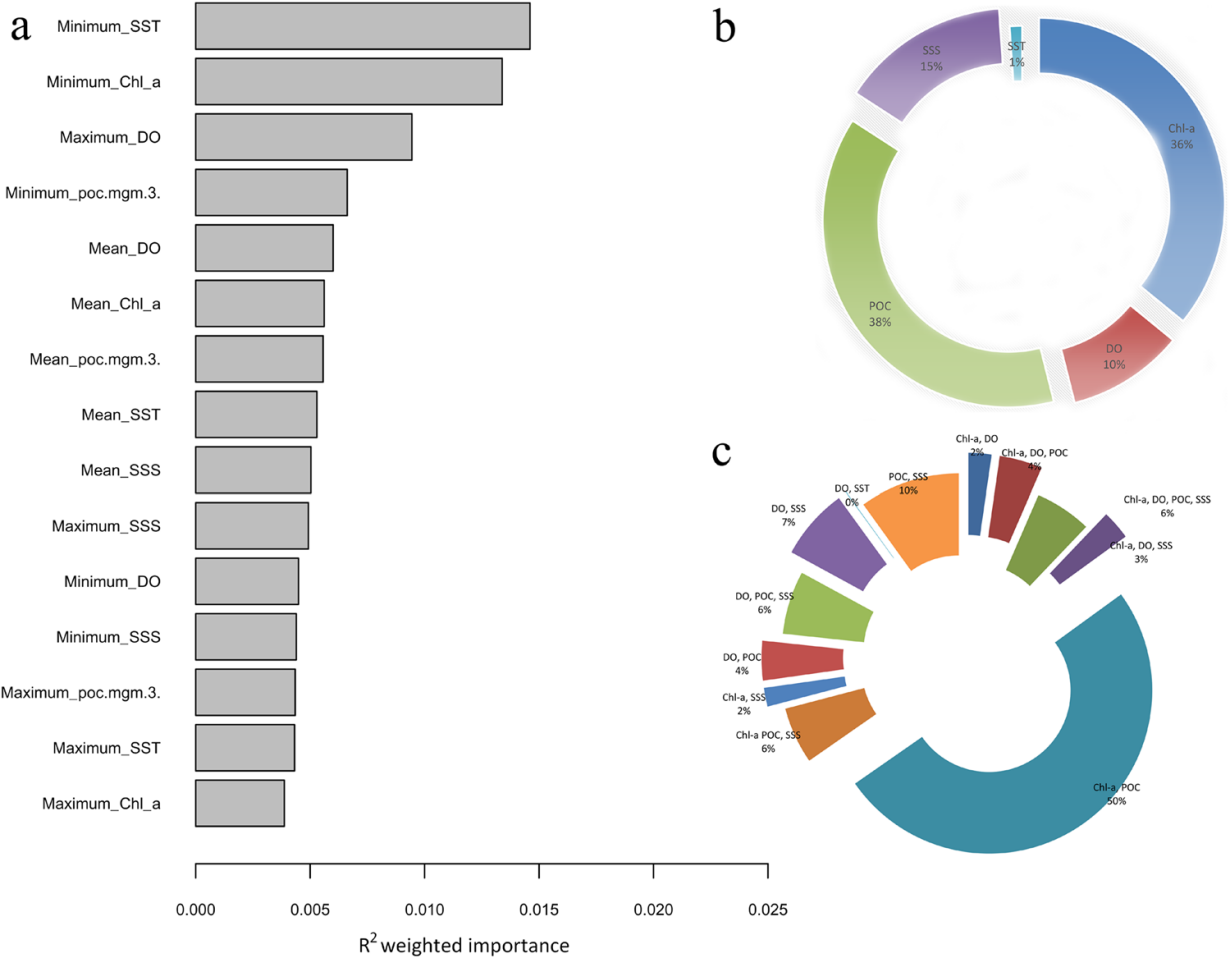
(**a**) The contribution of each environmental variable in explaining genetic variation across *S. longiceps* populations generated from gradient forest analysis. (**b**) The percentage of loci associated with chlorophyll-*a* concentration (Chl-*a*), particulate organic carbon concentration (POC), dissolved oxygen concentration (DO), sea surface salinity (SSS), and sea surface temperature (SST) identified by LFMM analyses and (**c**) their overlaps. The plot was generated with LFMM in LEA^48^.

The ‘snmf’ function in LEA indicated that the ancestral population, K = 3 was the best fit for the genotypic matrix (Supplementary Fig. S12). The gradient forest analysis showed that the environmental variables of greatest importance were related to minimum SST, minimum Chl-*a* and maximum dissolved oxygen—DO (Fig. 4a). After combining the results from five independent runs, the histogram of adjusted *p*-values (*q*) confirmed correct distribution as recommended in the LFMM manual (as expected, flat with a peak close to zero)^48^ (Supplementary Figs. S13 and S14). Significant association with environmental gradients was detected at 4371 loci (8.8%) by LFMM analysis and among that 3411 SNP loci were unique with 38%, 36%,15%, 10% and 1% of them associated with POC, Chl-*a*, SSS, DO and SST, respectively (Fig. 4b,c)*.* Among the rest of the SNP loci, 50% SNPs were associated with Chl-*a* and POC, 10% with POC and SSS (Fig. 4c). Among the LFMM identified SNP loci, three were also identified with BAYESCAN (FDR < 0.05). Pairwise comparison of genetic divergence (F_ST_) using only the 4371 SNP loci exhibiting significant association with environmental gradients also indicated a similar population structure as observed in the analysis of all polymorphic loci (Table 2; Supplementary Table S3).

Among the 4371 adaptive loci, only 516 (11.8%) recorded significant similarity to the known genes in the SwissProt database and they were characterized into 320 groups involving molecular function, cellular component and biological process. The adaptive loci encoded genes are mostly involved in cellular energy metabolism, transcription, cell growth and signalling (Supplementary Tables S4 and S5). Most of the candidate or outlier SNP loci (88.2%) did not match with known genes in the public database and they may be representing non-coding regions of the genome.

### Larval dispersal

Probable spawning grounds of Indian oil sardine and its larval dispersion along the Indian coast are not yet located. The spawning grounds were identified based on the occurrence of oozing spawners or planktonic eggs along the southwest coast of India^49^. The fecundity of oil sardine ranged from 12,631 to 75,000 eggs^49,50^. The newly hatched larva generally floats passively on the water surface as oil globule and buoyancy of the yolk helps them to float on the water. During the spawning period (June–September), the spawners with an average size of 19 cm appear in the coastal waters every year^42^ and spawning takes place at approximately 40–60 m depth^51^. Eggs of the oil sardine are pelagic, transparent and spherical with an average diameter of 1.4 mm^42^. The newly hatched larva generally floats passively on the surface of the water (oil globule and buoyancy of the yolk helps to floats over the water surface). Embryonic development of the oil sardine is completed within 24–48 h and larval duration is between 30 and 40 days^30^. The average length of the newly hatched larva is 2.75 mm and by the end of this first day, the oil globule in the yolk disappears^30,42^. The smallest size of oil sardine recruited to the fishery is ~ 6 cm size.

**Figure 5:**
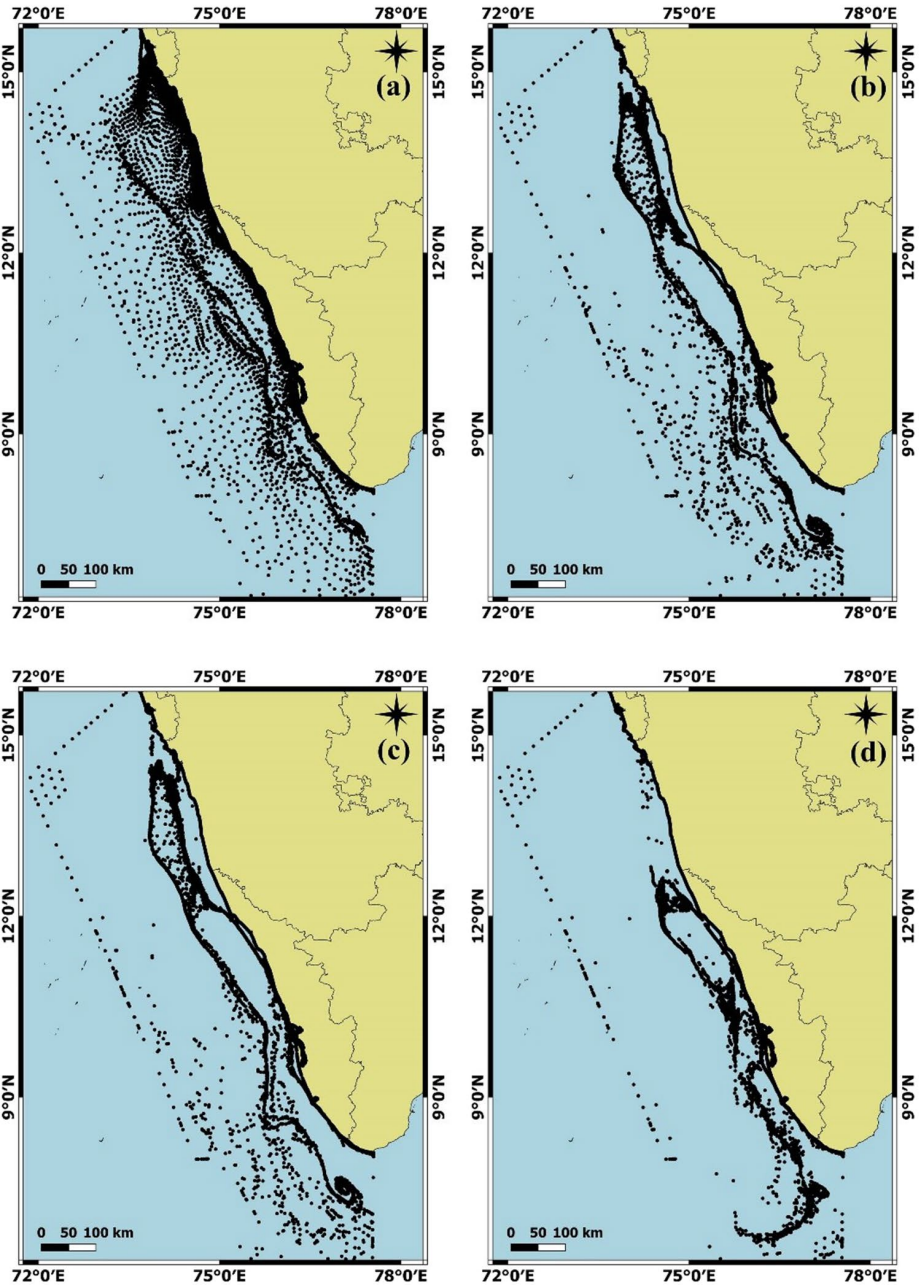
Larval dispersal along the southwest coast of India modelled using FVCOM (Finite volume community ocean model). Transportation of particles from initial release during July 2014 after (**a**) 1 day (**b**) 5 days (**c**) 10 days (**d**) 20 days. The plot was generated with GMSH software (https://gmsh.info/).

Simulation studies revealed the spatial variation of egg and larvae (passive particles) during July and January, which were shown in Fig. 5 and Supplementary Fig. S15, respectively. Three major regions were identified from the analysis during both seasons via, (1) transportation of particles close to the coastal region (2) transportation of particles through the shelf currents (3) circulation of particles due to the eddy off the southern tip of India. During January, the mass transportation of particles towards the north is identified (Supplementary Fig. S15). After 20 days, the majority of the particles were accumulated at the coastal waters of Kochi, northern Kerala and the Karnataka coastal region. While in July, the southwest coast of India experiences the monsoonal winds which generate the southward coastal currents. The larvae exactly achieve the momentum within one day and get transported towards the south with respect to the West Indian Coastal Current (WICC). The presence of particles at the Karnataka coast is up to 10 days only, after that the particles move further towards the southern side and accumulate within 20 days at the entire Kerala coast especially the coastal regions of Northern Kerala, Kochi, Alappuzha and Kollam (Fig. 5).

## Discussion

Genome-wide SNPs generated from ddRAD sequencing data indicated that Oman (OMAN) population has highly diverged from Indian populations (NEAS, SEAS, SBoB and NBoB) with significant F_ST_ values (0.065–0.075), which indicated reduced connectivity between these populations. On the contrary, no significant genetic differentiation was observed between populations along the Indian coast when F_ST_ values were analyzed (F_ST_ ranging from 0.0001 to 0.075). Yet, a low degree of genetic differentiation was observed between NEAS vs other populations from the Indian coast mainly SEAS, SBoB and NBoB in both PCA and the least-squares estimate of ancestry proportions. The large intra-population variation observed in NEAS might represent cryptic diversity that needs to be investigated further. Larval transport modelling with particle tracking method indicated the major role of wind and associated coastal currents in the dispersal of the larvae in the water column along the southwest coast of India. Wide variations in the monthly Chl-*a*, SST and SSS were noticed between coastal regions of OMAN, NEAS, SEAS, SBoB and NBoB of the Indian Ocean, and its correlation with oil sardine abundance is noticed. The association of the sardine fishery with sea surface temperature and chlorophyll-*a* observed in the present study corroborate the findings that successful sardine fishery is associated with the optimum values of these parameters (lower temperature (26–28 °C) and higher food (Chl-*a* < 5 mg/m^3^) availability during May–July). The shift in the habitat characteristics may be acting as environmental barriers for successful larval dispersal, persistence and genetic introgression (which would explain the neutral divergence). Fluctuations in the environmental parameters may induce selective pressure in the oil sardine populations of different eco-regions (adaptive divergence). The SST, Chl-*a*, and DO were the predominant factors explaining the observed genetic variation between Oman and the other Indian Ocean populations. Signals of local adaptation were present in Indian oil sardine populations and a set of loci associated with SST, Chl-*a* and DO were also identified in the analyses.

Genetic differentiation is considered to be proportional to the number of migrants in each generation. In a pelagic fish like oil sardine with large effective population size, high fecundity, long planktonic larval phase and high dispersal due to oceanic circulation, the effect of genetic drift is considered to be negligible on generating signals of genetic differentiation^52^. However, relatively higher levels of genetic diversity were observed in Indian oil sardine populations in the present study (π ranged from 0.0015 to 0.0028) than those observed in other marine fishes^53^. The average observed heterozygosity values using microsatellites were four times higher than those observed when using SNPs^31^. On the contrary, the genetic differentiation, quantified as pairwise F_ST_, was similar in both types of datasets. Comparable results were obtained in STRUCTURE analysis using microsatellite markers as the runs converged at K = 2 with low admixture between populations^31^, when Oman vs Indian Ocean populations was analyzed. Analyzing the microsatellite data omitting Oman samples illustrated a low level of genetic structuring between NEAS vs SEAS and BoB^31^. In the present study also, the maximum number of outlier loci were detected in comparisons between Oman and other Indian Ocean samples. On the contrary, a lower level of outlier loci was detected in comparisons between NEAS and SEAS, potentially indicating a low level of adaptive divergence between them.

Significant genetic differentiation between Oman and Indian populations of *S. longiceps* indicated restricted gene flow between these two regions, mainly contributed by the oceanographic and environmental barriers. A high level of variation in monthly SST, Chl-*a* and SSS was noticed between coastal regions of OMAN and NEAS of the Indian Ocean, which can act as environmental barriers for successful larval dispersal and persistence. Oceanographic barriers like currents and eddies also may be preventing successful larval dispersal and genetic introgression. Oman Sea is characterized by strong coastal upwelling along the western ocean boundary induced by the Somali Current and Findlater jet, and the Oman sardine populations are associated with this highly productive ecosystem. The northward flowing Somali Current (SC) during Southwest monsoon seasons can disperse larvae from OMAN to NEAS. However, the absence or discontinuous low-temperature zone and Chl-*a* zones between OMAN to SEAS (Gulf of Oman) may prevent the survival of larvae dispersed by SC. Besides, the presence of eddies like the Great Whirl generated by the Somali current and the Socotra Gyre observed in the Oman sea during the summer season^54^, coincides with the spawning time of Indian oil sardine which may act as larval retention zones creating restricted mixing and subsequent genetic differentiation.

Along the Indian coastline, a lack of significant genetic differentiation was observed in F_ST_ analyses whilst a low level of genetic differentiation between NEAS and other regions (SEAS, SBoB and NBoB) was observed in both PCA and Least-squares estimates of ancestry proportions. The intra-population divergence observed in NEAS in PCA analysis might indicate cryptic diversity which needs to be investigated further.

A significant number of private alleles were also observed in each population along the Indian coastline. Consistent with this observation, the study using microsatellite markers has revealed the occurrence of intra-population divergence in the *S. longiceps* populations along the Indian coastline with significant genetic differentiation between SEAS and NEAS^31^. This low level of genetic differentiation may be due to locally adapted regions of the genome because the low genetic divergence can be maintained even in the presence of geographical connectivity and gene flow, as selection and adaptive divergence can operate over micro-spatial scales^55^. The presence of outlier loci and the high number of private alleles in these populations corroborate these conclusions. The homogenising effect of the high larval dispersal rate in the water column along the southwest coast of India by wind and associated coastal currents, as observed in the larval transport modelling in the present study may be the reason for observed poor genetic differentiation signals between SEAS, SBoB and NBoB. We could identify only a few F_ST_ outliers in comparisons between NEAS and other regions (SEAS, SBoB and NBoB). This may be due to the stringent filtration method used by BAYEScan and the de novo method of analysis. Thus, to generate a complete picture of the selective forces acting on this species, a reference genome-based analysis (which can achieve higher statistical power in the outlier analyses) is needed. Higher density reference-based genome scans can reveal some additional candidate loci under strong or moderate selection in the *S. longiceps* populations that were not covered in the present study^56,57^. A zone of high temperature and low Chl-*a* is also existing between NEAS and SEAS till the end of southwest monsoon season (June–September) which also acts as an environmental barrier restricting larval dispersal and gene flow. Overall, the conclusions of the present study using RADseq agree with the findings regarding the genetic structuring of Indian oil sardine derived using mitochondrial and polymorphic microsatellite markers^31,32^. Similar levels of low but significant genetic differentiation have been reported in marine species like Marine snail^58^, European seabass^6^ and Amur ide^56^ using the ddRAD sequencing approach.

The observed genetic structuring in Indian oil sardine is due to the presence of barriers between eco-regions such as geographic distance, patchiness in the environment, local and global oceanic circulation patterns and environmental gradients which prevents gene flow to some extent^59,60^. The small pelagic fishery is associated with major upwelling regions which are the most productive in the world’s oceans and consequently their abundance is controlled by food availability^61^. The seasonal-annual fluctuations in the sardine fishery have been linked with fluctuations in productivity (Chl-*a*) of upwelling regions and changes in the environmental parameters such as temperature (SST)^29,44^. These two parameters have a significant role in the spawning of sardine and biological behaviour of early life stages (egg and yolk-sac larvae, early feeding larvae, late feeding larvae, juveniles) which has also been established from the present study. An optimum environmental window of temperature (lower temperature (26–28) during May—July), food (planktons, diatoms like *Fragilaria oceanica*^43^), salinity and dissolved oxygen along with other physicochemical ocean characters such as wind events, upwelling^37^ and tidal variability (which control the above environmental characters and larval dispersal) throughout early life stages is necessary for the survival of larvae and subsequent recruitment. Thus, shifts in habitat characteristics may not be acting strictly as a barrier to the migration of adults but may prevent successful spawning, larval dispersal and subsequent colonization^62^. The near optimum characteristics (lower temperature (26–28 °C) and higher food (Chl-*a*) availability during May–July) found in OMAN and SEAS make them the most productive regions. Fluctuations in the sardine landings from these regions were linked with deviations from the optimum habitat requirements (temperature and food).

The SEAS population is associated with the highly productive Malabar upwelling zone in the south-eastern boundary of the Arabian Sea, and a high level of connectivity and lack of genetic divergence has been observed between NEAS vs SBoB and NBoB. Oceanographic parameters along these regions may be providing a favourable condition for substantial gene flow and mixing between these regions. Seasonal reversals in the boundary currents mainly the West India Coastal Current (WICC) and the East India Coastal Current (EICC) along the coastal regions^46^ provide adequate opportunities for successful larval dispersal and subsequent colonization. Continuous transport of larvae from SEAS to BoB by WICC and EICC is likely responsible for the observed genetic connectivity between the two oceans. The continuous low temperature and high Chl-*a* zones along the southwest and southeast coast of India during spawning time can support the survival of larvae and subsequent colonization by providing an optimum environmental window of temperature and food. This could be one of the survival mechanisms of oil sardine in its heterogeneous and changing habitat.

Very little information is available regarding the dispersal of larvae and migratory potential or pattern of sardine post larvae. Sardine larvae are pelagic and planktonic with a larval duration of approximately 40 days^30^, but no information exists regarding their dispersal potential. The spawning season of Indian oil sardine coincides with the period of West India Coastal Current (WICC) that flows towards the south along the west coast of India and the East India Coastal Current (EICC) that flows towards the north during the southwest monsoon season^45^. The Lagrangian particle tracking method was widely used in larval transport in the Gulf of Maine^63^ and pollutant transport studies in the Cochin estuary^64^. The study by Seena et al.^65^ using the FVCOM model revealed that numerous rivers debouching freshwater to the coastal areas create buoyant plumes and transport in accordance with coastal currents. Larval transport modelling with particle tracking method in the present study indicated the major role of wind and associated coastal currents (WICC) in the dispersal of the larvae in the water column along the southwest coast of India. Besides larval dispersal, localized migration by adult sardine shoals may also contribute to gene flow and connectivity to some extent as sardine shoals are reported to swim at a speed of 5 km/h^37^.

The genetically differentiated *S. longiceps* from the Indian ocean can be considered as two locally adapted populations; the OMAN population adapted to a habitat with low temperature (24–27 °C) and high food availability (Chl-*a*) and the Indian coastal population (NEAS, SEAS, SBoB and NBoB) adapted to high temperature (26–30 °C) and low food availability (Chl-*a*). The illustrated genetic divergence (as measured by the F_ST_ values in Table S3) of loci associated with environmental parameters supports the presence of local adaptation. Furthermore, the association of candidate loci with environmental gradients suggests an important role of SST, Chl-*a* and DO concentration in the genetic structuring of *S. longiceps*. Most of the candidate loci identified were associated with cellular energy metabolism, transcription, cell growth and signalling potentially indicating the selective pressures to cope up with the heterogeneous oceanic realm. Analyses on selection on mitochondrial OXPHOS genes of *S. longiceps* also confirmed the importance of heterogenous oceanographic patterns induced by fluctuations in sea surface temperature, chlorophyll-*a* and dissolved oxygen concentration on mitogenomic protein-coding genes and several adaptive mitogenomic loci were identified by the present authors^32^. Additionally, previous studies have reported divergent morphology in sardines from OMAN (heavier with greater body depth) and Indian coastal regions (two morphotypes mainly stout and slender)^34^. These morphotypes may be the result of divergent selection and adaptive variation which corroborates our findings.

Local adaptation may prevent successful colonization and persistence of recruits from non-matching environments due to the competitive advantage of adapted populations^66^. These natal habitats will also be important for the aggregation of spawners from locally adapted populations which may induce natal philopatry and subsequent genetic differentiation. Spawning aggregations and natal philopatry have been reported in many fish species^67,68^. Local adaptation is expected in species with restricted gene flow^4,69^, but recent investigations have provided evidence for selection in high gene flow species^70–72^ like Indian oil sardine. Adaptation and selection can occur even in the presence of high gene flow as genomic regions associated with selection may be tightly linked where recombination does not take place as adaptive alleles may exhibit hitchhiking and consequent divergent selection^8^. An important benefit of high-density marker loci like SNPs generated by RADseq is the possibility of locating the genomic regions with high population structuring which may restrict gene flow^73^. The loci identified as F_ST_ outliers with F_IS_ > 0 may be the representation of genomic regions of local adaptation, isolated genomic regions of divergence with gene flow, and genomic regions of speciation^74–76^ in *S. longiceps*. Thus, the signals of cryptic structuring or assortative mating can be used as a starting point for a more detailed study to identify the genomic region of genetic divergence in *S. longiceps* and Clupeoids. Reanalysis of the RADseq data with a reference genome-based method is necessary for identifying genome-wide distribution or chromosomal regions of genetic divergence. Locally adapted populations correlating with habitat heterogeneity may be providing resilience to environmental fluctuations as biological complexity and cryptic diversity acts as buffering mechanisms. Larval dispersal and population connectivity across homogeneous habitats like Indian coastal regions may provide additional benefits to an abundant population to survive and persist.

Climate change is capable of altering the ecological interactions, inducing physiological stress, and bringing about changes in phenology and seasonal timing^77^. Knowledge regarding genetic structure at a spatial and temporal scale can be used to monitor climatic impacts as shifts in the stable structuring can happen due to climate change-induced localized extinctions and recolonizations^78^. Extirpation of local populations causes irreversible changes in the gene pool, impairing the potential for adaptive evolution^69,79^. Besides, when locally adapted populations or cryptic diversity decline, immigration cannot replenish or rehabilitate declining populations as non-matching habitats will induce negative selection^69,79^. Recent evidence regarding recruitment failure and decline in sardine populations due to global warming and overfishing, especially along the Malabar upwelling zone emphasizes the vital need for preserving the adaptive potential of exploited species. The present study is the first attempt in these lines and the findings of the present study are very relevant for devising management and conservation measures to augment the biodiversity of natural stocks of Indian oil sardine which otherwise may decline to the point of no recovery due to the threat of warming of oceans and overfishing. The two major locally adapted subpopulations need to be managed separately to ensure the preservation of biodiversity (spawning components), which is crucial for climatic resilience^68^. Range expansion of Indian oil sardines to northern latitudes has been reported^80^ which in due course of time may result in range shifts and localized extinctions especially along Malabar upwelling zones as oceans of the lower latitudes will be more prone to the perils of ocean warming. We suggest managing Indian oil sardine region wise by seasonal closures and mesh size regulations to prevent further deterioration of natural stocks. Management and conservation of this vital resource capable of providing a cheap source of protein for the millions in developing countries like India are very important for ensuring sustainability.

## Materials and methods

### Sample collection and DNA extraction

**Figure 6:**
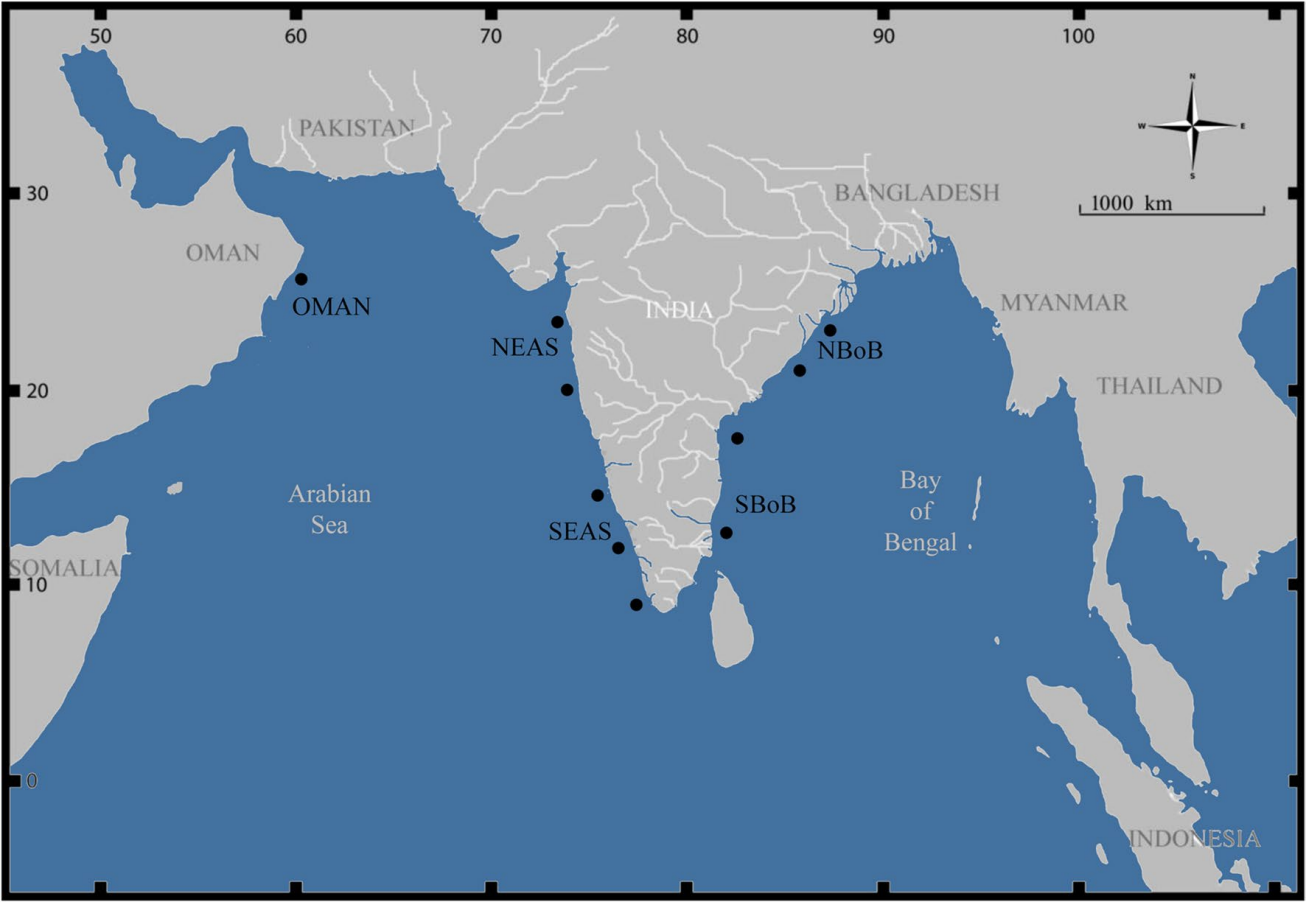
Ecoregions or sampling sites of *Sardinella longiceps*. *OMAN* Oman Sea, *NEAS* North East Arabian Sea, *SEAS* South East Arabian Sea, *SBoB* South West Bay of Bengal, *NBoB* Northwest Bay of Bengal. Black dots indicate sampling points in each ecoregion. The map was drawn using Adobe Photoshop CS6 (https://www.adobe.com/in/products/photoshop.html?promoid=PC1PQQ5T&mv=other).

A total of 100 *Sardinella longiceps* samples were collected from the five eco-regions of the Indian Ocean, mainly, Oman Sea (OMAN), North Eastern Arabian Sea (NEAS), South Eastern Arabian Sea (SEAS), Southern Bay of Bengal (SBoB), and Northern Bay of Bengal (NBoB) during 2014–2017 (Fig. 6). Tissue samples collected from mature individuals (stage IV) were stored in 95% ethanol at − 20 °C for genomic DNA extraction. Genomic DNA was extracted using DNAeasy blood and tissue kit (Qiagen) and quality was visualized on a 0.8% agarose gel and quantified with NanoDrop One (Thermo Fisher Scientific) and Qubit 3.0 Fluorometer (Thermo Fisher Scientific). The fishes sampled in this study were collected from government-approved fishing vessels as indicated in the guidelines for the care and use of wild-caught fish in research by De Tolla et al.^81^. The protocols were approved by the ethical committee of the ICAR-Central Marine Fisheries Research Institute, Kochi. The methods are also reported in accordance with ARRIVE guidelines (https://arriveguidelines.org).

### ddRAD library construction, sequencing and SNP genotyping

ddRAD sequencing was carried out using the high-quality DNA extracted from each sample (20 samples each from OMAN, NEAS, SEAS, SBoB and NBoB). The ddRAD libraries were prepared based on the protocol by Peterson et al.^14^. The DNA of each sample was double digested completely with high-fidelity *MspI* and *EcoRI* restriction enzymes (New England Biolabs). The barcode with a unique 5-bp sequence and P1 adapter was ligated to *EcoRI* overhang and the P2 adapter was ligated to *MspI* overhang. The DNA fragments were selected on an automated size selection technology BluePippin (Sage Science) with a mean size of 300 bp on a 2% agarose cartridge. The fragments were then PCR amplified and purified with AMPure XP Beads. Libraries were prepared with approximately equal amounts of DNA from each sample. The barcoded ddRAD libraries were sequenced on an Illumina HiSeq 2500 platform with a 2 × 100 bp sequencing approach.

The raw reads were demultiplexed with a specific barcode index and filtered using the 'process_radtags’ program in STACKS v1.40^82^. The quality of the demultiplexed reads was assessed using FastQC^83^, reads with low quality (Phred score < 30) and uncalled bases were discarded and the sequences were trimmed to 85 bp. Identification of SNPs and genotype calling were performed in STACKS using ‘denovo_map.pl’ program. ‘ustacks’ (-m,4) constructed stacks for each sample, ‘cstacks’ (-M,5; -n,6) used all individuals from each population to construct a catalog of loci and 'sstacks' compared each sample against the catalog. No reference genome was available for this species during analysis. The number of SNPs was used to optimize the values of three main parameters -m, -M, -N of ‘ustacks’ and -n of 'csstacks’, as described by Paris et al.^84^. We used 50 samples with the greatest depth of coverage to optimize combinations of different parameter values by setting -N as M + 1 and increasing parameters (-M, -N and -n) until it reached a plateau. The number of SNPs and percentage of polymorphic loci reached a plateau at a combination of -m = 4, -M = 5 and -n = 6 and it was selected as optimal for analyzing the entire data set. Then individuals with more than 20% missing data were removed and polymorphic RAD loci with 1SNP and 2alleles were retained. Finally, loci that deviated from the Hardy–Weinberg equilibrium (HWE) in a single population were removed.

### Estimation of genome-wide genetic diversity and differentiation

We used different methods to analyze genetic diversity and patterns of genetic structuring within our dataset. Population genetic statistics (allele frequencies, percentage of polymorphic loci, nucleotide diversity, Wright’s F-statistics, the number of nucleotide sites across each population data set, percentage polymorphic sites and the average frequency of the major allele (P) at the sites) were computed using 'population’ program in STACKS v1.40. We used one random SNP per locus, the minimum number of populations in which a locus must be present to process a locus (–min-populations) as 5, the minimum percentage of individuals in a population required to process a locus for that population (–min-samples-per-pop) as 90% and minimum minor allele frequency required to process a nucleotide site at a locus (–min_maf) as 2%. The global estimate for genetic differentiation was assessed using F_ST_ and R_ST_ values in GENEPOP v4.0^85^.

STRUCTURE v2.3^86^ with a model-based Bayesian MCMC clustering was used to determine the number of genetically distinct populations (K) with the highest posterior probability. K values ranging from 1 to 5 were simulated under the admixture model to determine the optimal K value. The program was run for a burn-in period of 100,000 followed by 500,000 MCMC steps. ∆K method^40^ was used to detect the optimal K value. To identify the presence of any hierarchical structure, the analysis was repeated after excluding the cluster identified in the first run.

We performed a principal component analysis (PCA) of full data with R package Adegenet v2.1.2^87^ to visualize broad-scale population structure. A neighbour-joining method, clustering of the population as implemented in Neighbor (from Phylip programs)^88^ was used to generate a phylogenetic tree using average pairwise F_ST_ values as input. The tree was then visualized in FigTree^89^. A simple mantel test using F_ST_ and spatial distance matrix (shortest sea route between the sampling sites) was performed with zt^90^. A regression analysis with pairwise F_ST_/(1 − F_ST_) and log of the pairwise spatial distance between populations^91^ was also carried out.

We used BAYESCAN v2.1^92^ to identify loci under divergent selection, based on the differences in the allele frequencies between populations. The allele frequencies of each locus in each population or input file were prepared by converting GENEPOP files to BAYESCAN input files using PGDSpider v2.1.1.5^93^. The SNP loci with false discovery rate (FDR) < 0.05 were selected as outlier SNPs and all other optional parameters were set as default. The result was plotted on a graph with the R statistical package.

### Fish landing and annual climatology data

We analyzed the long-term catch data of oil sardine with climatology data to understand the relationship between fluctuations in sardine abundance, distribution and climatology from the five eco-regions of the present study. Annual landing data of Indian oil sardine along the east and west coast of India was analyzed (CMFRI 1985–2019) for a period of 34 years from 1985 to 2019. Monthly sea surface temperature (SST °C) data were extracted from NOAA, Physical Science Laboratory (https://psl.noaa.gov/data/gridded/data.noaa.oisst.v2.highres.html) and chlorophyll-*a* (Chl-*a* mg/m^3^) L3 data from MODIS site (https://modis.gsfc.nasa.gov/data/dataprod/chlor_a.php), for a period of 18 years from 2002 to 2020. In addition to surface climatology data, we also analyzed water column data (2014–2017) such as SST, sea surface salinity (SSS ppt) and dissolved oxygen (DO μmol/kg) from World Ocean Database available at https://www.nodc.noaa.gov/OC5/woa18/woa18data.html and Chl-*a* (mg/m^3^) L3 data from MODIS site (https://modis.gsfc.nasa.gov/data/dataprod/chlor_a.php). The data sets were analyzed using open-source software Ferret and visualized in Ocean Data View (ODV 5.1.7). For each eco-region, the annual minimum, maximum and mean of each of these environmental factors were estimated and used to test the environmental variable that explains the genetic variation among the *S. longiceps* population samples, using the gradient forests R package^94^.

### Detection of SNP loci associated with environmental variables

The association between SNPs and climate gradients was tested using the latent factor mixed model (LFMM) in LEA^48^ to detect signals of local adaptation. This method analyses the SNP allele-environment correlation between each SNP and environmental variable by correcting the background population structure. We calculated the individual admixture coefficients from the genotypic matrix using the 'snmf' function, estimated the entropy criterion and chose the number of ancestral populations (K) that best explained the genotypic data^48^. Five independent LFMM runs were conducted using 100,000 iterations with a burn-in of 10,000 and calculated the median Z-score (which is the strength of genetic-environmental association) for each locus. Adjusted *p*-values (q) were calculated using the false discovery rate (FDR) method and the histogram of q inspected as recommended in the LFMM manual^48^. SNP loci with q < 0.05 (or FDR < 0.05) were classified as candidate loci. Each candidate or outlier SNP loci that contained putatively adaptive regions were subjected to a BLASTx search of all sequences in the SwissProt, RefSeq protein and NCBI (non-redundant database, e-value = 10). GO Annotator (http://xldb.di.fc.ul.pt/rebil/tools/goa/.) was used for assisting the GO annotation of loci that produced significant blast hits.

### Larval dispersal

Larval dispersal along the southwest coast of India was modelled using FVCOM (Finite volume community ocean model)^95^, a validated hydrodynamic model^65^ coupled with a particle tracking module^64^ during January 2014 (Winter monsoon) and July 2014 (Summer monsoon). GMSH software (https://gmsh.info) was used to generate an unstructured grid that consisted of 27,264 nodes and 48,089 elements with 21 vertical sigma layers. The model domain extended from 6°N (Kanyakumari) to 15°N (Goa) along the Southwest coast of India with a spatial resolution range of 10 m (near to the coast and islands) to 50 km (Open Ocean boundary). The open boundary of the model was forced with the tide (FES2014), salinity, temperature and sea surface height (HYCOM data). The daily river discharge data (26 rivers) during the year 2014 collected from the Central water commission of India was included in the model. The model was forced by the surface meteorological parameters such as atmospheric pressure, air temperature, wind, heat flux and precipitation taken from the European Centre for Medium Weather Forecast (ECMWF). Fifty thousand passive particles were introduced as neutrally buoyant at the surface layer in the entire model domain node and recoded its path at 1-h intervals during January and July (Supplementary Fig. S16).

## Supplementary Information

Supplementary Information.

## Data Availability

Data used in this study are available on request from the authors.
