# Supplementary material for: Genomic investigations provide insights into the mechanisms of resilience to heterogeneous habitats of the Indian Ocean in a pelagic fish
Source: Sci Rep. 2021 Oct 19;11:20690. doi: 10.1038/s41598-021-00129-5 (PMC8526693; doi:10.1038/s41598-021-00129-5)
Supplement: Supplementary file 1 — Supplementary Information. [file 41598_2021_129_MOESM1_ESM.pdf]

Genomic investigations provide insights into the mechanisms of resilience to heterogeneous habitats of the Indian Ocean in a pelagic fish

Wilson Sebastian[1*], Sandhya Sukumaran[1], S. Abdul Azeez[2], K. R. Muraleedharan[2], P.K. Dinesh Kumar[2], P.U. Zacharia[1], A. Gopalakrishnan[1]

[1]Marine Biotechnology Division, Central Marine Fisheries Research Institute, Ernakulam North P.O., Kochi – 682018, Kerala, India.

[2]CSIR-National Institute of Oceanography, Regional Centre Kochi, Dr Salim Ali Road, Post Box No. 1913, Kochi, 682018, Kerala, India.

Table S1 Summary genetic statistics for restriction-site associated DNA (RAD) sites of *S. longiceps*

Nb loci genotyped	**56,358**
Nb loci genotyped by at least 50% of individuals	**53,680**
Nb polymorphic loci genotyped	**49,361**
Nb polymorphic loci genotyped by at least 50% of individuals	**48,473**
Nb polymorphic loci with 1 SNP and 2 alleles	**50,076**

Table S2 Summary of zygosity of *S. longiceps* samples used for restriction-site associated DNA (RAD) analysis

S No	Sample	Population id	Missing genotype	Heterozygote genotype	Homozygote genotype	Heterozygosity rate (%)
1	OMAN1M	OMAN	8,263	8,395	39,700	17.46
2	OMAN3M	OMAN	56,339	1	18	5.26
3	OMAN4M	OMAN	7,769	8,399	40,190	17.29
4	OMAN5M	OMAN	7,440	9,011	39,907	18.42
5	OMAN5Ma	OMAN	7,794	8,606	39,958	17.72
6	OMAN6M	OMAN	10,379	5,492	40,487	11.94
7	OMAN7	OMAN	20,097	3,538	32,723	9.76
8	OMAN8	OMAN	15,812	3,176	37,370	7.83
9	OMAN9	OMAN	6,188	7,861	42,39	15.67
10	OMAN10	OMAN	13,159	3,809	39,390	8.82
11	OMAN11	OMAN	8,954	7,670	39,734	16.18
12	OMAN12	OMAN	11,983	3,937	40,438	8.87
13	BOM1	NEAS	8,988	5,329	42,041	11.25
14	BOM2	NEAS	5,361	8,317	42,680	16.31
15	BOM3	NEAS	6,568	7,857	41,933	15.78
16	MALV11	NEAS	16,737	3,040	36,581	7.67
17	MALV12	NEAS	12,583	4,554	39,221	10.4
18	MALV2	NEAS	32,781	3,579	19,998	15.18
19	MALV5	NEAS	12,018	5,075	39,265	11.45
20	MALV7	NEAS	9,023	8,531	38,804	18.02
21	MALV8	NEAS	19,320	2,379	34,659	6.42
22	MALV9	NEAS	15,482	3,398	37,478	8.31
23	MANG	NEAS	34,981	1,252	20,125	5.86
24	MUM1Fa	NEAS	11,754	8,319	36,285	18.65
25	MUM5Ma	NEAS	10,658	8,392	37,308	18.36
26	COH1	SEAS	15,812	3,176	37,370	7.83
27	COH10	SEAS	8,772	5,696	41,890	11.97
28	COH1Ma	SEAS	8,988	5,329	42,041	11.25
29	COH2	SEAS	6,338	7,900	42,120	15.79
30	COH2Fa	SEAS	8,774	5,724	41,860	12.03
31	COH3	SEAS	5,021	9,447	41,890	18.4

32	COH3F	SEAS	36,304	339	19,715	1.69
33	COH3Fa	SEAS	6,950	7,899	41,509	15.99
34	COH3Ma	SEAS	6,557	7,918	41,883	15.9
35	COH4	SEAS	5,591	8,791	41,976	17.32
36	COH5	SEAS	6,279	7,599	42,480	15.17
37	COH5Ma	SEAS	8,874	5,600	41,884	11.79
38	COH6	SEAS	6,314	7,696	42,348	15.38
39	COH7	SEAS	6,188	7,869	42,301	15.68
40	COH8	SEAS	7,210	6,768	42,380	13.77
41	COH9	SEAS	5,960	7,977	42,421	15.83
42	KNR2	SEAS	19,135	3,785	33,438	10.17
43	KNR3	SEAS	13,159	3,809	39,390	8.82
44	KNR4	SEAS	6,045	7,807	42,506	15.52
45	KNR5	SEAS	8,938	5,527	41,893	11.66
46	KNR6	SEAS	7,088	6,729	42,541	13.66
47	KNR8	SEAS	5,898	8,354	42,106	16.56
48	KNR9	SEAS	5,766	8,391	42,201	16.59
49	VIZ1	SEAS	7,231	6,855	42,272	13.95
50	VIZ2	SEAS	6,221	7,961	42,176	15.88
51	VIZ3	SEAS	5,361	9,567	41,430	18.76
52	VIZ4	SEAS	6,697	7,571	42,090	15.25
53	MADPM1	SBoB	8,793	5,508	42,057	11.58
54	MADPM10	SBoB	5,468	8,559	42,331	16.82
55	MADPM12	SBoB	8,954	7,670	39,734	16.18
56	MADPM13	SBoB	12,549	3,980	39,829	9.08
57	MADPM2	SBoB	9,016	7,790	39,552	16.45
58	MADPM3	SBoB	7,897	6,104	42,357	12.6
59	MADPM4	SBoB	15,479	2,896	37,983	7.08
60	MADPM5	SBoB	5,853	8,214	42,291	16.26
61	MADPM6	SBoB	5,622	9,155	41,581	18.04
62	MADPM8	SBoB	11,983	3,937	40,438	8.87
63	MADPM9	SBoB	13,243	3,526	39,589	8.18
64	MAND1M	SBoB	7,645	6,298	42,415	12.93
65	MAND2F	SBoB	6,568	7,857	41,933	15.78
66	MAND2M	SBoB	6,236	8,498	41,624	16.95
67	MAND15	SBoB	8,988	5,329	42,041	11.25
68	MAND14	SBoB	5,846	7,807	42,705	15.46
69	ODIS1	NBoB	4,917	9,002	42,439	17.5
70	ODIS10	NBoB	8,938	5,527	41,893	11.66
71	ODIS9	NBoB	7,210	6,768	42,380	13.77
72	ODIS8	NBoB	5,766	8,391	42,201	16.59
73	ODIS6	NBoB	5,468	8,559	42,331	16.82
74	ODIS11	NBoB	7,416	6,644	42,298	13.58
75	ODIS12	NBoB	4,852	8,967	42,539	17.41
76	ODIS13	NBoB	5,846	7,807	42,705	15.46
77	ODIS2	NBoB	5,386	9,422	41,550	18.48
78	ODIS3	NBoB	7,259	6,489	42,610	13.22
79	ODIS4	NBoB	4,437	9,995	41,926	19.25
80	ODIS5	NBoB	5,361	8,317	42,680	16.31
81	ODIS7	NBoB	5,250	9,412	41,696	18.42
82	ODIS8	NBoB	5,000	9,658	41,700	18.81
83	VSKP	NBoB	16,513	4,182	35,663	10.5

OMAN-Oman Sea, NESA-North East Arabian sea, SEAS-South East Arabian sea, SBOB-South West Bay of Bengal, NBOB-Northwest Bay of Bengal.

Table S3 The pairwise comparison of genetic distance (F_{ST}) among *S. longiceps* populations using 4371 SNP loci significantly associated with environmental gradients

Population	OMAN	NESA	SEAS	SBOB	NBOB
OMAN	0	Highly sign.	Highly sign.	Highly sign.	Highly sign.
NESA	0.0756	0	sign.	sign.	sign.
SEAS	0.0656	0.0004	0	Not sign.	sign.
SBOB	0.0674	0.0005	0.0003	0	Not sign.
NBOB	0.0681	0.0012	0.0034	0.0007	0

OMAN-Oman Sea, NESA-North East Arabian Sea, SEAS-South East Arabian Sea, SBOB-South West Bay of Bengal, NBOB-Northwest Bay of Bengal.

Table S4 Summary of GO Terms for adaptive loci of *S. longiceps* from the Indian Ocean

S NO	GO Terms	Aspect	
1	1,4-alpha-glucan branching enzyme activity	F Molecular Function	enables
2	acetylcholine-gated cation-selective channel activity	F	enables
3	acetylgalactosaminyl-O-glycosyl-glycoprotein beta-1,6-N-acetylglucosaminyltransferase activity	F	enables
4	actin binding	F	enables
5	actin cytoskeleton organization	P Biological Process	involved_in
6	actin filament binding	F	enables
7	amino acid transport	P	involved_in
8	androgen receptor binding	F	enables
9	arterial endothelial cell differentiation	P	involved_in
10	Atg1/ULK1 kinase complex	C Cellular Component	part_of
11	ATP binding	F	enables
12	ATP transmembrane transporter activity	F	enables
13	ATP transport	P	involved_in
14	atrioventricular valve morphogenesis	P	involved_in
15	biological_process	P	acts_upstream_of_or_within
16	blood circulation	P	involved_in
17	blood vessel morphogenesis	P	involved_in
18	calcium ion binding	F	enables
19	carbohydrate metabolic process	P	involved_in
20	carbohydrate phosphorylation	P	involved_in
21	carboxylic acid metabolic process	P	involved_in
22	cardiolipin binding	F	enables
23	catalytic activity	F	enables
24	cation binding	F	enables
25	cation transmembrane transport	P	involved_in
26	cation transmembrane transporter activity	F	enables
27	cation transport	P	involved_in
28	cell adhesion	P	involved_in
29	cell differentiation	P	involved_in
30	cell junction	C	part_of
31	cell maturation	P	acts_upstream_of_or_within
32	cell projection	C	part_of
33	cell surface receptor signaling pathway	P	involved_in
34	cellular component organization	P	involved_in
35	cellular glucose homeostasis	P	involved_in
36	cellular iron ion homeostasis	P	involved_in
37	cellular metabolic process	P	involved_in
38	cellular protein modification process	P	involved_in
39	cellular response to DNA damage stimulus	P	involved_in
40	cellular response to drug	P	involved_in
41	cellular response to estrogen stimulus	P	involved_in
42	cellular response to progesterone stimulus	P	involved_in
43	central nervous system myelination	P	involved_in
44	centriole replication	P	involved_in
45	centriole-centriole cohesion	P	involved_in

46	chaperone-mediated protein complex assembly	P	involved_in
47	chromatin	C	part_of
48	chromatin binding	F	enables
49	cis-regulatory region sequence-specific DNA binding	F	enables
50	cohesin complex	C	part_of
51	collagen trimer	C	part_of
52	cone photoresponse recovery	P	involved_in
53	copper ion binding	F	enables
54	cysteine-type peptidase activity	F	enables
55	cytoplasm	C	part_of
56	cytoplasmic microtubule	C	part_of
57	cytoskeleton organization	P	involved_in
58	cytosol	C	part_of
59	Derlin-1 retrotranslocation complex	C	part_of
60	developmental growth	P	acts_upstream_of_or_within
61	digestive tract development	P	involved_in
62	digestive tract morphogenesis	P	acts_upstream_of_or_within
63	DNA binding	F	enables
64	DNA duplex unwinding	P	involved_in
65	DNA helicase activity	F	enables
66	DNA integration	P	involved_in
67	DNA metabolic process	P	involved_in
68	DNA recombination	P	involved_in
69	DNA repair	P	involved_in
70	DNA topoisomerase type II (double strand cut, ATP-hydrolyzing) activity	F	enables
71	DNA topological change	P	involved_in
72	DNA-binding transcription activator activity	F	enables
73	DNA-binding transcription activator activity, RNA polymerase II-specific	F	enables
74	DNA-binding transcription factor activity	F	enables
75	DNA-binding transcription factor activity, RNA polymerase II-specific	F	enables
76	dorsal aorta development	P	involved_in
77	dynein complex	C	part_of
78	early endosome	C	part_of
79	embryonic viscerocranium morphogenesis	P	involved_in
80	endoplasmic reticulum	C	part_of
81	endoplasmic reticulum membrane	C	part_of
82	endosome	C	part_of
83	enteric nervous system development	P	involved_in
84	ERAD pathway	P	acts_upstream_of_or_within
85	estrogen receptor binding	F	enables
86	excitatory postsynaptic potential	P	involved_in
87	extracellular ligand-gated ion channel activity	F	enables
88	extracellular matrix	C	part_of
89	extracellular matrix organization	P	involved_in
90	extracellular matrix structural constituent	F	enables
91	extracellular region	C	part_of
92	extracellular space	C	part_of
93	extrinsic component of mitochondrial inner membrane	C	part_of
94	extrinsic component of mitochondrial outer membrane	C	part_of
95	FAD binding	F	enables
96	fat cell differentiation	P	involved_in
97	ferroxidase activity	F	enables
98	flavin adenine dinucleotide binding	F	enables
99	G protein-coupled receptor activity	F	enables
100	G protein-coupled receptor kinase activity	F	enables
101	G protein-coupled receptor signaling pathway	P	involved_in
102	G1/S transition of mitotic cell cycle	P	involved_in
103	glucose binding	F	enables
104	glycogen biosynthetic process	P	involved_in
105	glycolytic process	P	involved_in
106	glycylpeptide N-tetradecanoyltransferase activity	F	enables
107	Golgi apparatus	C	part_of
108	GTP binding	F	enables
109	GTPase activator activity	F	enables
110	GTPase activity	F	enables
111	guanyl-nucleotide exchange factor activity	F	enables

112	guanyl-nucleotide exchange factor complex	C	part_of
113	heart development	P	involved_in
114	heart looping	P	involved_in
115	helicase activity	F	enables
116	hematopoietic progenitor cell differentiation	P	involved_in
117	heme transmembrane transporter activity	F	enables
118	heme transport	P	involved_in
119	hexokinase activity	F	enables
120	histone acetylation	P	involved_in
121	histone acetyltransferase activity	F	enables
122	histone acetyltransferase complex	C	part_of
123	histone methylation	P	involved_in
124	histone methyltransferase activity	F	enables
125	host cell nucleus	C	part_of
126	Hrd1p ubiquitin ligase ERAD-L complex	C	part_of
127	hyaluronic acid binding	F	enables
128	hydrolase activity	F	enables
129	hydrolase activity, hydrolyzing O-glycosyl compounds	F	enables
130	identical protein binding	F	enables
131	immunoglobulin production in mucosal tissue	P	involved_in
132	in utero embryonic development	P	acts_upstream_of_or_within
133	inositol phosphate biosynthetic process	P	involved_in
134	integral component of membrane	C	part_of
135	intestinal absorption	P	involved_in
136	inward rectifier potassium channel activity	F	enables
137	ion channel activity	F	enables
138	ion transmembrane transport	P	involved_in
139	ion transport	P	involved_in
140	iron ion transport	P	involved_in
141	isomerase activity	F	enables
142	kidney morphogenesis	P	involved_in
143	kinase activity	F	enables
144	kinetochore binding	F	enables
145	kinetochore microtubule	C	part_of
146	lacrimal gland development	P	acts_upstream_of_or_within
147	ligase activity	F	enables
148	liver development	P	involved_in
149	L-lactate dehydrogenase activity	F	enables
150	magnesium ion binding	F	enables
151	maintenance of blood-brain barrier	P	involved_in
152	melanocyte differentiation	P	acts_upstream_of_or_within
153	membrane	C	part_of
154	metal ion binding	F	enables
155	methylation	P	involved_in
156	methyltransferase activity	F	enables
157	microtubule motor activity	F	enables
158	microtubule plus-end binding	F	enables
159	microtubule-based movement	P	involved_in
160	microtubule-based process	P	involved_in
161	mitochondrial inner membrane	C	part_of
162	mitochondrial outer membrane	C	part_of
163	mitochondrion	C	part_of
164	mitotic sister chromatid cohesion	P	involved_in
165	monooxygenase activity	F	enables
166	morphogenesis of a branching epithelium	P	acts_upstream_of_or_within
167	morphogenesis of an epithelium	P	involved_in
168	motor activity	F	enables
169	mRNA binding	F	enables
170	multicellular organism development	P	involved_in
171	myosin complex	C	part_of
172	negative regulation of apoptotic process	P	involved_in
173	negative regulation of autophagosome assembly	P	involved_in
174	negative regulation of canonical Wnt signaling pathway	P	involved_in
175	negative regulation of gene expression	P	involved_in
176	negative regulation of macroautophagy	P	involved_in
177	negative regulation of neurogenesis	P	involved_in

178	negative regulation of NF-kappaB transcription factor activity	P	involved_in
179	negative regulation of protein kinase activity	P	involved_in
180	negative regulation of Schwann cell proliferation	P	acts_upstream_of_or_within
181	negative regulation of transcription by RNA polymerase II	P	involved_in
182	negative regulation of transcription, DNA-templated	P	involved_in
183	neural crest cell migration	P	involved_in
184	neural plate development	P	involved_in
185	neuronal stem cell population maintenance	P	involved_in
186	Notch signaling pathway	P	involved_in
187	nucleic acid binding	F	enables
188	nucleoplasm	C	part_of
189	nucleotide binding	F	enables
190	nucleus	C	part_of
191	oligodendrocyte development	P	involved_in
192	oligodendrocyte differentiation	P	involved_in
193	oxidation-reduction process	P	involved_in
194	oxidoreductase activity	F	enables
195	oxidoreductase activity, acting on paired donors, with incorporation or reduction of molecular oxygen, NAD(P)H as one donor, and incorporation of one atom of oxygen	F	enables
196	oxidoreductase activity, acting on paired donors, with incorporation or reduction of molecular oxygen, reduced flavin or flavoprotein as one donor, and incorporation of one atom of oxygen	F	enables
197	oxidoreductase activity, acting on the CH-OH group of donors, NAD or NADP as acceptor	F	enables
198	peptidase activity	F	enables
199	peptidyl-arginine methylation	P	involved_in
200	peptidyl-arginine N-methylation	P	involved_in
201	peripheral nervous system development	P	involved_in
202	peripheral nervous system neuron axonogenesis	P	involved_in
203	peroxisomal importomer complex	C	part_of
204	peroxisomal membrane	C	part_of
205	peroxisome	C	part_of
206	peroxisome proliferator activated receptor binding	F	enables
207	pharyngeal system development	P	involved_in
208	phosphatidylinositol-3,4,5-trisphosphate binding	F	enables
209	phosphorylation	P	involved_in
210	phosphotransferase activity, alcohol group as acceptor	F	enables
211	photoreceptor activity	F	enables
212	photoreceptor cell maintenance	P	involved_in
213	photoreceptor inner segment	C	part_of
214	phototransduction, visible light	P	involved_in
215	plasma membrane	C	part_of
216	plasma membrane organization	P	involved_in
217	positive regulation of apoptotic process	P	involved_in
218	positive regulation of autophagosome maturation	P	involved_in
219	positive regulation of cysteine-type endopeptidase activity involved in apoptotic process	P	involved_in
220	positive regulation of gene expression	P	acts_upstream_of_or_within
221	positive regulation of gliogenesis	P	involved_in
222	positive regulation of GTPase activity	P	involved_in
223	positive regulation of myelination	P	acts_upstream_of_or_within
224	positive regulation of neuroblast proliferation	P	acts_upstream_of_or_within
225	positive regulation of nucleic acid-templated transcription	P	involved_in
226	positive regulation of TOR signaling	P	involved_in
227	positive regulation of transcription by RNA polymerase II	P	involved_in
228	positive regulation of transcription, DNA-templated	P	involved_in
229	postsynaptic membrane	C	part_of
230	potassium ion import across plasma membrane	P	involved_in
231	potassium ion transmembrane transport	P	involved_in
232	potassium ion transport	P	involved_in
233	potassium transmembrane transporter activity, phosphorylative mechanism	F	enables
234	potassium:proton exchanging ATPase activity	F	enables
235	progesterone receptor binding	F	enables
236	promoter-specific chromatin binding	F	enables

237	pronephros development	P	involved_in
238	protein binding	F	enables
239	protein deubiquitination	P	involved_in
240	protein folding	P	involved_in
241	protein homodimerization activity	F	enables
242	protein import into peroxisome matrix, docking	P	involved_in
243	protein kinase activity	F	enables
244	protein kinase binding	F	enables
245	protein kinase inhibitor activity	F	enables
246	protein phosphorylation	P	involved_in
247	protein secretion	P	involved_in
248	protein serine/threonine kinase activity	F	enables
249	protein transport	P	involved_in
250	protein ubiquitination	P	involved_in
251	protein-arginine N-methyltransferase activity	F	enables
252	proteolysis	P	involved_in
253	proton transmembrane transport	P	involved_in
254	Rab guanyl-nucleotide exchange factor activity	F	contributes_to
255	regulation of androgen receptor signaling pathway	P	involved_in
256	regulation of autophagy	P	involved_in
257	regulation of blood vessel diameter	P	involved_in
258	regulation of cell cycle	P	involved_in
259	regulation of cell morphogenesis	P	involved_in
260	regulation of developmental process	P	involved_in
261	regulation of ion transmembrane transport	P	involved_in
262	regulation of postsynaptic membrane potential	P	involved_in
263	regulation of receptor-mediated endocytosis	P	involved_in
264	regulation of TORC1 signaling	P	involved_in
265	regulation of transcription by RNA polymerase II	P	involved_in
266	regulation of transcription, DNA-templated	P	involved_in
267	regulation of vascular permeability	P	involved_in
268	response to bacterium	P	involved_in
269	response to endoplasmic reticulum stress	P	acts_upstream_of_or_within
270	retinoic acid receptor binding	F	enables
271	retrograde protein transport, ER to cytosol	P	acts_upstream_of_or_within
272	Rho GTPase binding	F	enables
273	rhodopsin kinase activity	F	enables
274	rhombomere boundary formation	P	involved_in
275	ribosome	C	part_of
276	RNA binding	F	enables
277	RNA polymerase II cis-regulatory region sequence-specific DNA binding	F	enables
278	RNA-dependent DNA biosynthetic process	P	involved_in
279	RNA-directed DNA polymerase activity	F	enables
280	sclerotome development	P	involved_in
281	semaphorin receptor binding	F	enables
282	sequence-specific DNA binding	F	enables
283	signal transduction	P	involved_in
284	signaling receptor activity	F	enables
285	signaling receptor binding	F	enables
286	sister chromatid cohesion	P	involved_in
287	somatic stem cell population maintenance	P	involved_in
288	spliceosomal complex assembly	P	involved_in
289	stem cell differentiation	P	involved_in
290	structural constituent of ribosome	F	enables
291	symmetric cell division	P	involved_in
292	synapse	C	part_of
293	telomere maintenance	P	involved_in
294	thiol-dependent ubiquitinyl hydrolase activity	F	enables
295	thyroid hormone receptor binding	F	enables
296	tissue morphogenesis	P	involved_in
297	transcription coactivator activity	F	enables
298	transcription coregulator activity	F	enables
299	transcription elongation from RNA polymerase II promoter	P	acts_upstream_of_or_within
300	transcription factor binding	F	enables
301	transcription regulatory region sequence-specific DNA binding	F	enables
302	transferase activity	F	enables

303	transferase activity, transferring acyl groups	F	enables
304	transferase activity, transferring glycosyl groups	F	enables
305	translation	P	involved_in
306	transmembrane signaling receptor activity	F	enables
307	transmembrane transport	P	involved_in
308	transmembrane transporter activity	F	enables
309	triglyceride metabolic process	P	involved_in
310	ubiquinone biosynthetic process	P	involved_in
311	ubiquitin-dependent ERAD pathway	P	involved_in
312	ubiquitin-dependent protein catabolic process	P	involved_in
313	ubiquitin-protein transferase activity	F	enables
314	unfolded protein binding	F	enables
315	ventral spinal cord interneuron differentiation	P	involved_in
316	ventriculo bulbo valve morphogenesis	P	involved_in
317	visual perception	P	involved_in
318	voltage-gated ion channel activity	F	enables
319	zinc ion binding	F	enables

F - Molecular Function, P - Biological Process, C - Cellular Component

Table S5 The species distribution summary of 4371 SNP loci significantly associated with environmental gradients in the Indian Ocean generated from BLASTx

S N0	Species Name
1	*Acanthochromis polyacanthus*
2	*Acinetobacter baumannii*
3	*Acropora digitifera*
4	*Ailuropoda melanoleuca*
5	*Amazona aestiva*
6	*Amphiamblys sp. WSBS2006*
7	*Amphiprion ocellaris*
8	*Anas platyrhynchos*
9	*Anopheles darlingi*
10	*Aotus nancymaae*
11	*Apostichopus japonicus*
12	*Aptenodytes forsteri*
13	*Aquila chrysaetos canadensis*
14	*Astyanax mexicanus*
15	*Austrofundulus limnaeus*
16	*Beggiatoa sp. 4572_84*
17	*Beggiatoa sp. PS*
18	*Bemisia tabaci*
19	*Boleophthalmus pectinirostris*
20	*Bos indicus*
21	*Bubalus bubalis*
22	*Buceros rhinoceros silvestris*
23	*Calidris pugnax*
24	*Callipepla squamata*
25	*Callorhinchus milii*
26	*Calypte anna*
27	*Camelus ferus*
28	*Candidatus Entotheonella sp. TSY2*
29	*Canis lupus familiaris*
30	*Cathartes aura*
31	*Cebus capucinus imitator*
32	*Cervus elaphus hippelaphus*
33	*Channa striata*
34	*Chelonia mydas*
35	*Chenopodium quinoa*
36	*Chinchilla lanigera*
37	*Chrysemys picta bellii*
38	*Ciona intestinalis*
39	*Clupea harengus*
40	*Columba livia*
41	*Corvus brachyrhynchos*
42	*Crassostrea gigas*

43	*Crassostrea virginica*
44	*Cricetulus griseus*
45	*Crocodylus porosus*
46	*Cuculus canorus*
47	*Cynoglossus semilaevis*
48	*Cyprinodon variegatus*
49	*Cyprinus carpio*
50	*Daboia russelii*
51	*Danio rerio*
52	*Dasypus novemcinctus*
53	*Dendroctonus ponderosae*
54	*Dicentrarchus labrax*
55	*Echinops telfairi*
56	*Emys marmorata pallida*
57	*Enhydra lutris kenyoni*
58	*Eptesicus fuscus*
59	*Equus caballus*
60	*Erinaceus europaeus*
61	*Esox lucius*
62	*Exaiptasia pallida*
63	*Folsomia candida*
64	*Fukomys damarensis*
65	*Fundulus heteroclitus*
66	*Galendromus occidentalis*
67	*Gallus gallus*
68	*Gavialis gangeticus*
69	*Gekko japonicus*
70	*Halyomorpha halys*
71	*Haplochromis burtoni*
72	*Heterocephalus glaber*
73	*Heteropneustes fossilis*
74	*Hippocampus comes*
75	*Homo sapiens*
76	*Horstia sp. AD1229*
77	*Hydra vulgaris*
78	*Hypophthalmichthys nobilis*
79	*Ictalurus punctatus*
80	*Ictidomys tridecemlineatus*
81	*Ixodes scapularis*
82	*Kryptolebias marmoratus*
83	*Labrus bergylta*
84	*Lachancea mirantina*
85	*Larimichthys crocea*
86	*Lasius niger*
87	*Lates calcarifer*
88	*Latimeria chalumnae*
89	*Lepidothrix coronata*
90	*Lepisosteus oculatus*
91	*Leptonychotes weddellii*
92	*Leptosomus discolor*
93	*Limosa lapponica baueri*
94	*Limulus polyphemus*
95	*Lonchura striata domestica*
96	*Lottia gigantea*
97	*Loxodonta africana*
98	*Macaca fascicularis*
99	*Macaca mulatta*
100	*Macaca nemestrina*
101	*Mandrillus leucophaeus*
102	*Marchantia polymorpha subsp. ruderalis*
103	*Maylandia zebra*
104	*Megachile rotundata*
105	*Meriones unguiculatus*
106	*Merops nubicus*
107	*Methylobacterium sp. 174MFSha1.1*
108	*Microtus ochrogaster*

109	*Miniopterus natalensis*
110	*Mizuhopecten yessoensis*
111	*Monopterus albus*
112	*Mus caroli*
113	*Mus musculus*
114	*Mus pahari*
115	*Mustela putorius furo*
116	*Myotis brandtii*
117	*Myotis davidii*
118	*Myotis lucifugus*
119	*Nannospalax galili*
120	*Nanorana parkeri*
121	*Natrix tessellata*
122	*Neolamprologus brichardi*
123	*Neotoma lepida*
124	*Nicrophorus vespilloides*
125	*Nomascus leucogenys*
126	*Nothobranchius furzeri*
127	*Notothenia coriiceps*
128	*Ochotona princeps*
129	*Octopus bimaculoides*
130	*Odobenus rosmarus divergens*
131	*Olea europaea var. sylvestris*
132	*Oncorhynchus kisutch*
133	*Oncorhynchus mykiss*
134	*Ooceraea biroi*
135	*Opisthocomus hoazin*
136	*Opisthorchis viverrini*
137	*Orbicella faveolata*
138	*Orcinus orca*
139	*Oreochromis niloticus*
140	*Ornithorhynchus anatinus*
141	*Orussus abietinus*
142	*Orycteropus afer afer*
143	*Oryctolagus cuniculus*
144	*Oryzias latipes*
145	*Pan troglodytes*
146	*Pantholops hodgsonii*
147	*Paralichthys olivaceus*
148	*Parasteatoda tepidariorum*
149	*Patagioenas fasciata monilis*
150	*Pelecanus crispus*
151	*Pelodiscus sinensis*
152	*Peromyscus maniculatus bairdii*
153	*Phalacrocorax carbo*
154	*Physeter catodon*
155	*Plecoglossus altivelis*
156	*Poecilia formosa*
157	*Poecilia latipinna*
158	*Poecilia mexicana*
159	*Poecilia reticulata*
160	*Pogona vitticeps*
161	*Priapulus caudatus*
162	*Protobothrops mucrosquamatus*
163	*Pseudomyrmex gracilis*
164	*Pseudopodoces humilis*
165	*Pundamilia nyererei*
166	*Pygocentrus nattereri*
167	*Python bivittatus*
168	*Rana catesbeiana*
169	*Rattus norvegicus*
170	*Rhincodon typus*
171	*Rhinolophus sinicus*
172	*Rhinopithecus roxellana*
173	*Salmo salar*
174	*Sarcophilus harrisii*

175	*Scleropages formosus*
176	*Scomber japonicus*
177	*Seriola dumerili*
178	*Seriola lalandi dorsalis*
179	*Sinocyclocheilus anshuiensis*
180	*Sinocyclocheilus grahami*
181	*Sinocyclocheilus rhinocerous*
182	*Sorex araneus*
183	*Spinacia oleracea*
184	*Stegastes partitus*
185	*Stomoxys calcitrans*
186	*Strongylocentrotus purpuratus*
187	*Struthio camelus australis*
188	*Stylophora pistillata*
189	*synthetic construct*
190	*Taeniopygia guttata*
191	*Takifugu rubripes*
192	*Tenualosa ilisha*
193	*Tetraodon nigroviridis*
194	*Thamnophis sirtalis*
195	*Thraustotheca clavata*
196	*Tinamus guttatus*
197	*Tribolium castaneum*
198	*Trichinella britovi*
199	*Trichinella nelsoni*
200	*Trichinella sp. T9*
201	*Trichuris suis*
202	*Tuber melanosporum Mel28*
203	*Tupaia chinensis*
204	*Tursiops truncatus*
205	*Tyto alba*
206	*ubiquinone*
207	*Ursus maritimus*
208	*Vicugna pacos*
209	*Vollenhovia emeryi*
210	*Xenopus laevis*
211	*Xenopus tropicalis*
212	*Xiphophorus maculatus*

Table S6 Alpha value of outlier loci potentially subjected to differential selection detected using BAYESCAN in genome-wide SNPs of *S. longiceps* populations.

Locus id	alpha	F_{ST}
All populations		
1	2.3143	0.27
2	1.9396	0.21
3	1.7974	0.20
4	1.7799	0.19
5	1.7123	0.18
OMAN vs Indian Ocean (NEAS, SEAS, SBoB, NBoB) population		
6	1.6756	0.27
7	2.0109	0.22
8	1.9829	0.21
Arabian Sea (NEAS, SEAS) and Bay of Bengal (SBoB, NBoB) population		
9	2.2139	0.07

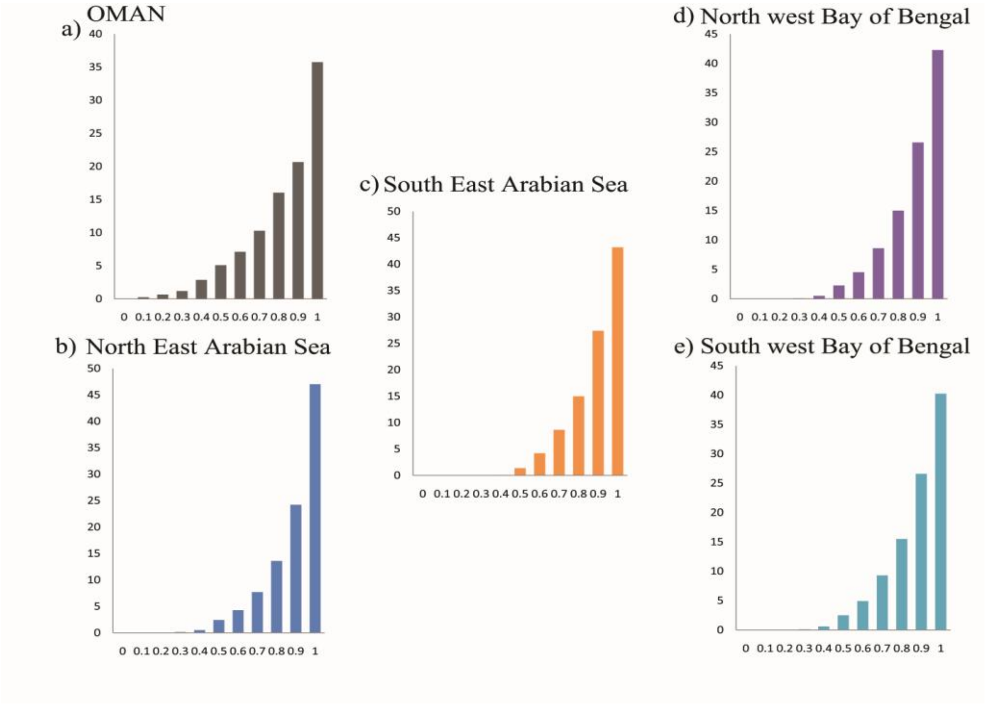

Figure S1 Allele frequency spectrum distribution for loci among *S. longiceps* populations.

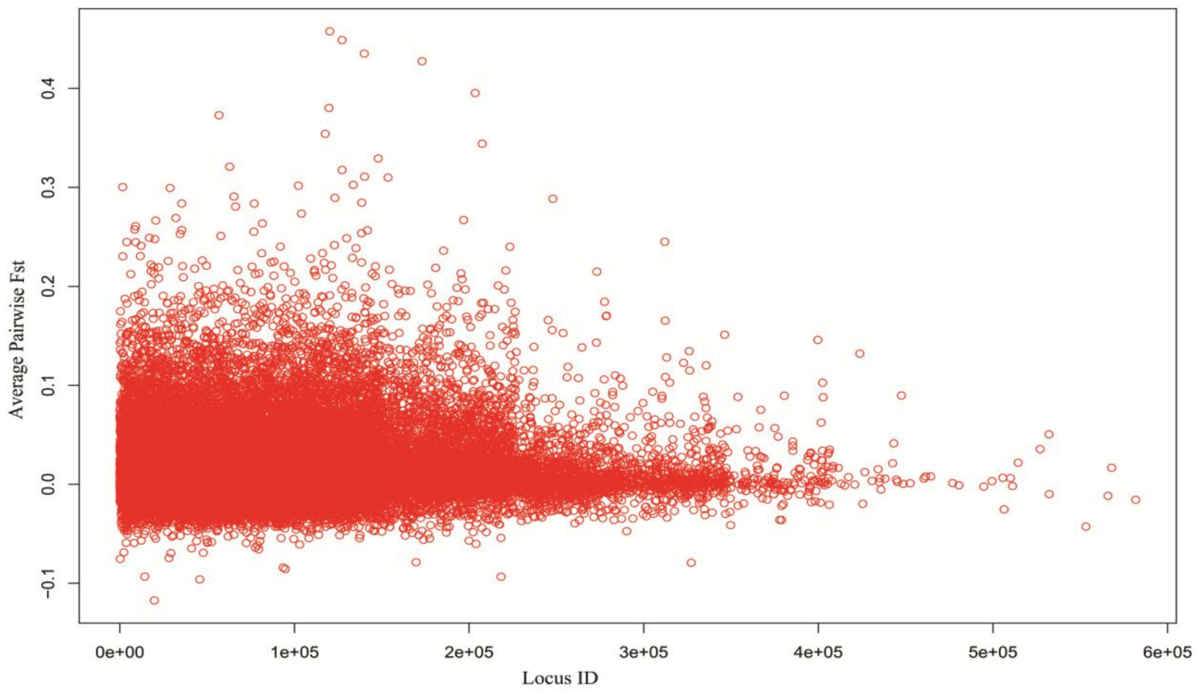

Figure S2 The plot of average pairwise F_{ST} of 56,358 SNPs loci between *S. longiceps* population. The x-axis represents the number of ID for each locus and the Y-axis indicates the pairwise F_{ST} values.

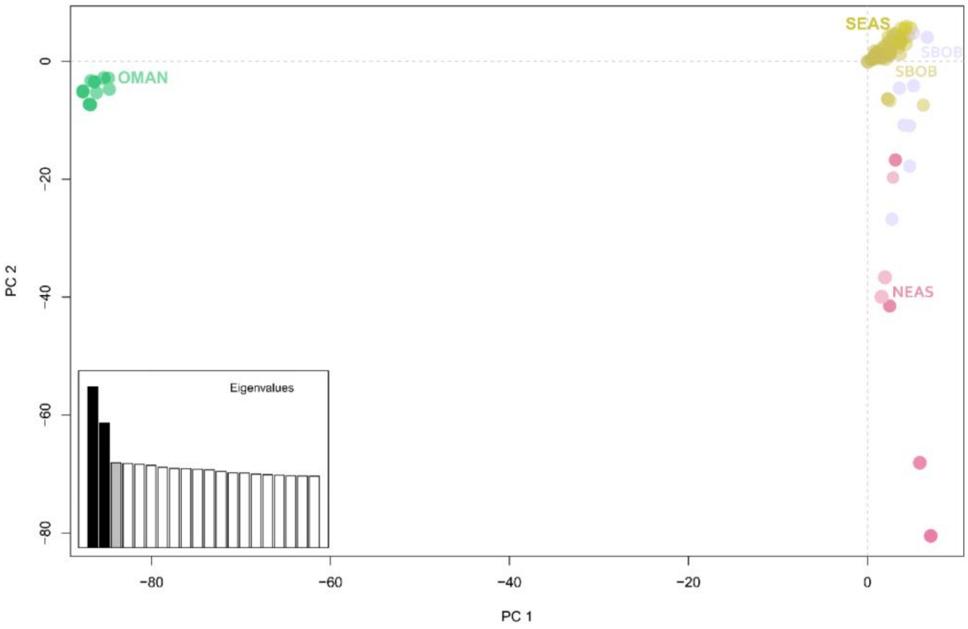

Figure S3 The scatter plot indicating individual variation in principal component (PC) scores derived from principal component analysis (PCA) of the *S. longiceps* RADseq data. Populations from different geographical regions (colour-coded) are represented as Oman Sea (OMAN), Northeast Arabian Sea (NEAS), Southeast Arabian Sea (SEAS), South West Bay of Bengal (SBoB) and Northwest Bay of Bengal (NBoB).

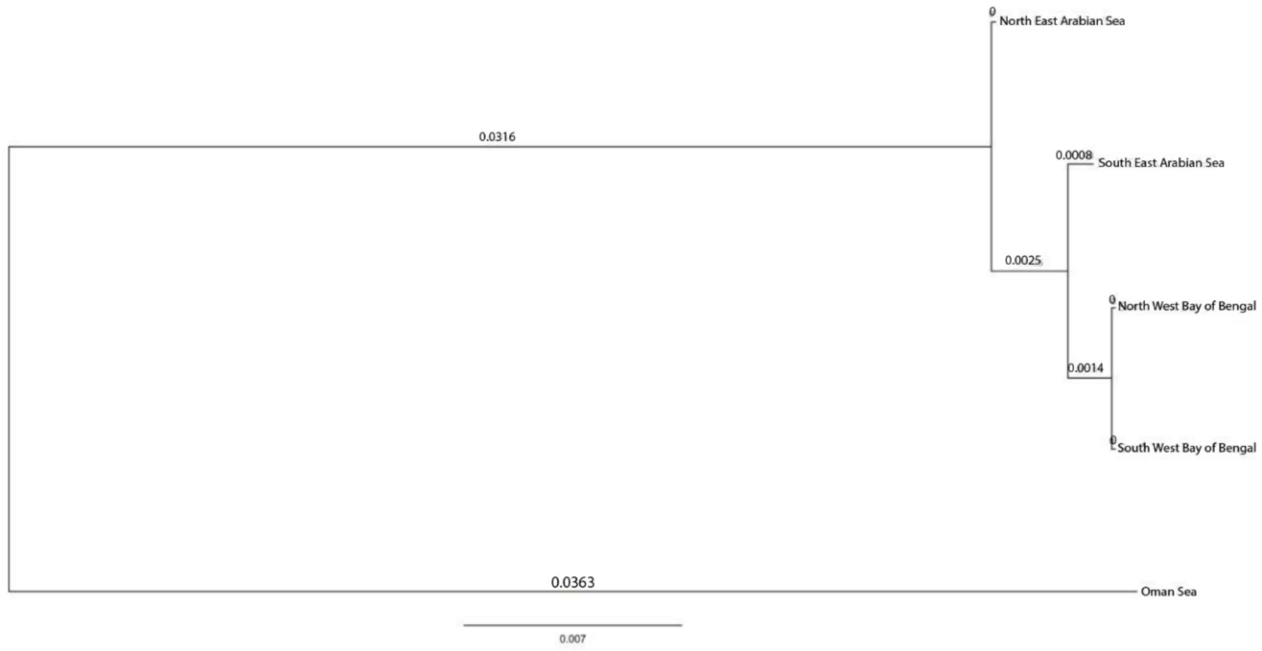

Figure S4 NJ tree of populations based on average F_{ST} values of 56,358.00 SNPs loci. Populations from different geographical regions are represented as Oman Sea, Northeast Arabian Sea, Southeast Arabian Sea, South West Bay of Bengal and Northwest Bay of Bengal.

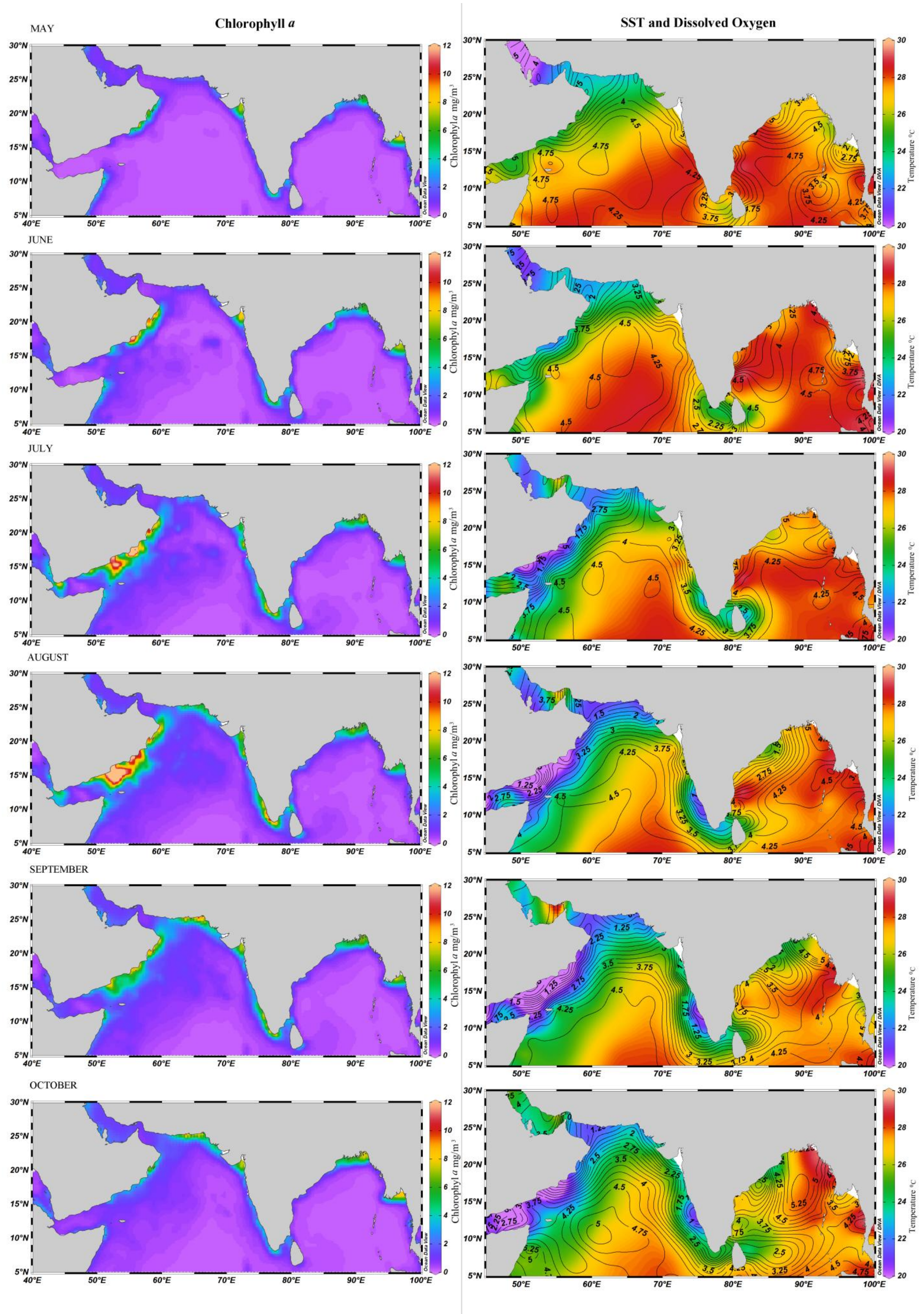

Figure S5 Monthly Chlorophyll *a* (mg/m^3), Sea Surface Temperature- SST (^{0}C) and Dissolved Oxygen (µmol/kg) for the Bay of Bengal and Arabian sea during May - October. Chlorophyll-*a* and Sea Surface Temperature gradients are represented as coloured shades. Dissolved Oxygen is represented as contour lines. The images were generated in ODV 5.1.7 (https://odv.awi.de/).

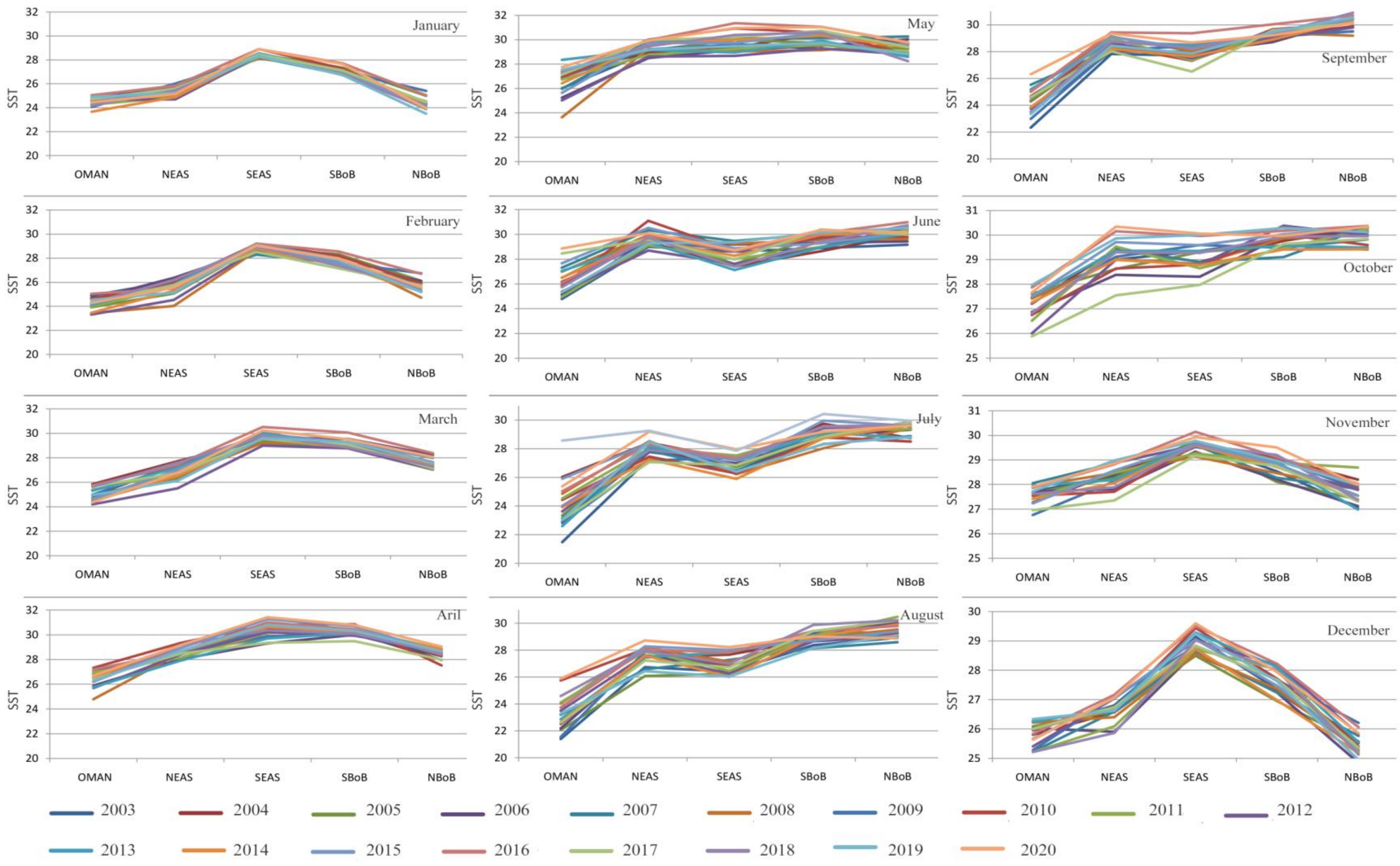

Figure S6 Monthly average Sea Surface Temperature (^{o}C) in the five ecoregions (OMAN, NEAS, SEAS, SBoB and NBoB) during 2003-2020. The X-axis indicates five ecoregions (OMAN-Oman Sea, NEAS-North East Arabian Sea, SEAS-South East Arabian Sea, SBOB-South West Bay of Bengal, NBOB-Northwest Bay of Bengal) and Y-axis indicates Sea Surface Temperature (SST) in ^{o}C.

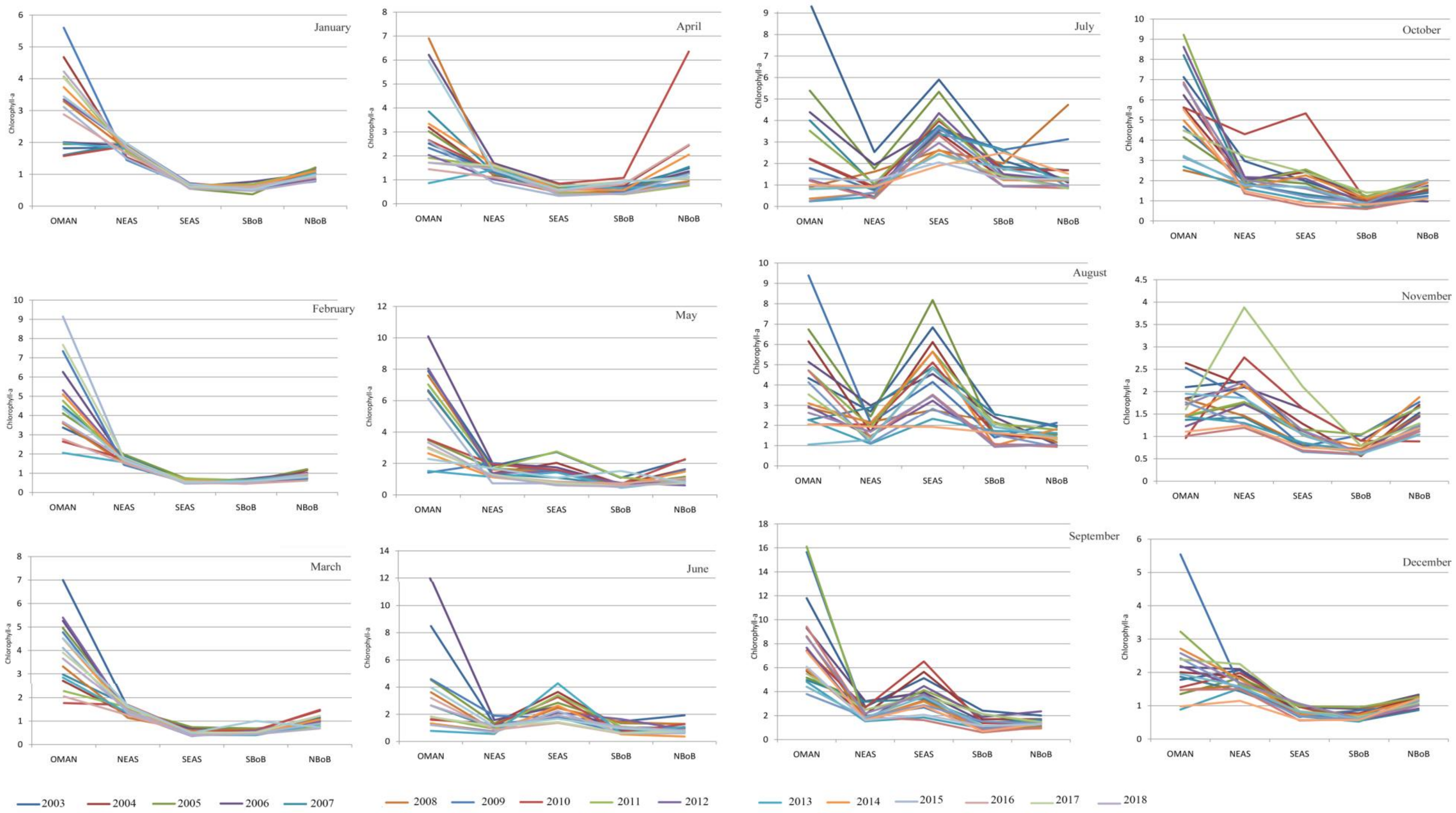

Figure S7 Monthly average Chlorophyll *a* (mg/m^3) in the five ecoregions (OMAN, NEAS, SEAS, SBoB and NBoB) during 2003-2018. The X-axis indicates five ecoregions (OMAN-Oman Sea, NESA-North East Arabian Sea, SEAS-South East Arabian Sea, SBOB-South West Bay of Bengal, NBOB-Northwest Bay of Bengal) and Y-axis indicates Monthly average Chlorophyll *a* in mg/m^3.

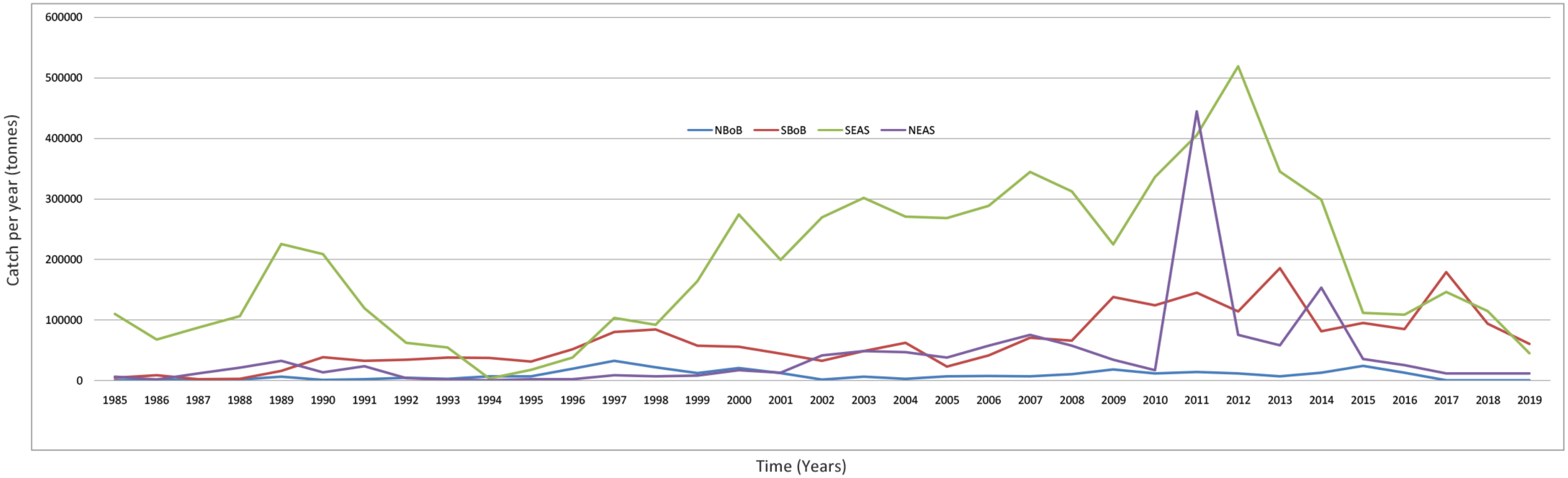

Figure S8 Variability in the estimated *S. longiceps* landing from NEAS, SEAS, SBoB and NBoB from 1985 to 2019. OMAN-Oman Sea, NESA-North East Arabian Sea, SEAS-South East Arabian Sea, SBOB-South West Bay of Bengal, NBOB-Northwest Bay of Bengal. The X-axis indicates the time in years and Y-axis indicates fish catch in tonnes.

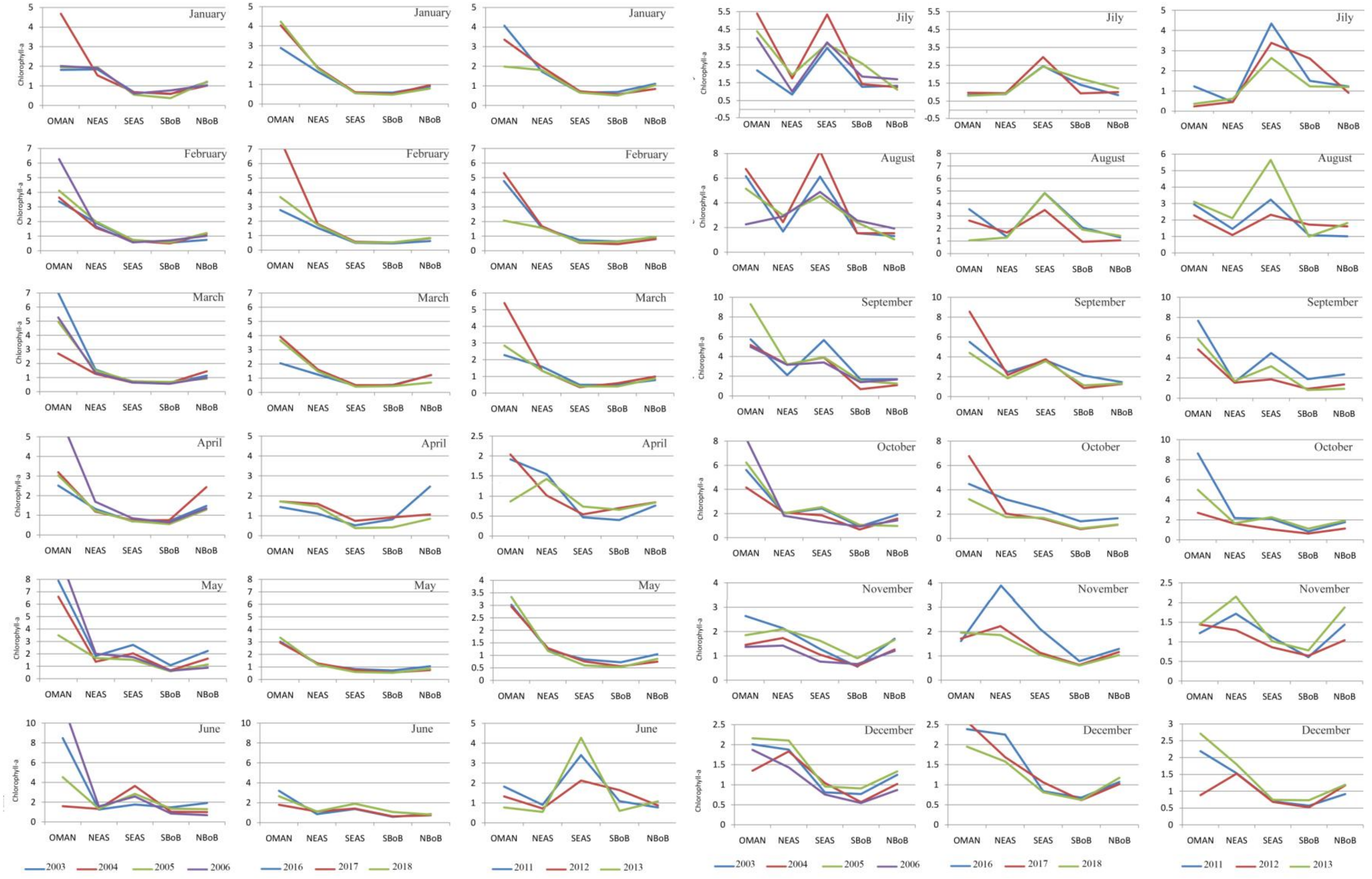

Figure S9 Monthly average Chlorophyll *a* (mg/m^3) in the five ecoregions (OMAN, NEAS, SEAS, SBoB and NBoB) during years associated with the lowest (2016-2018), intermediate (2002-2006) and abundant fishery (2011-2012). The X-axis indicates five ecoregions (OMAN-Oman Sea, NEAS-North East Arabian sea, SEAS-South East Arabian sea, SBOB-South West Bay of Bengal, NBOB-Northwest Bay of Bengal) and Y-axis indicates Monthly average Chlorophyll *a* in mg/m^3.

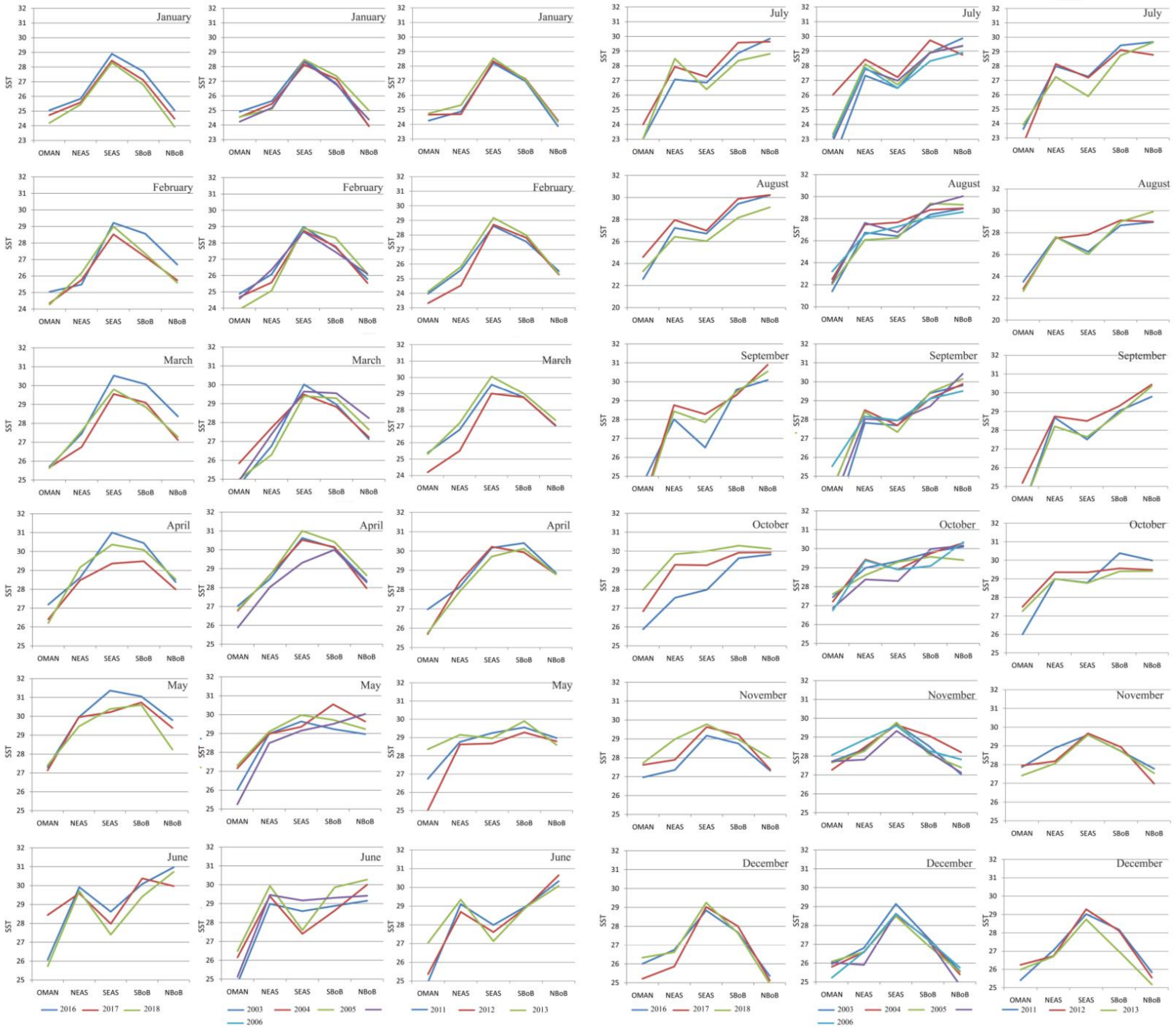

Figure S10 Monthly average Sea Surface Temperature (^oC) in the five ecoregions (OMAN, NEAS, SEAS, SBoB and NBoB) during years associated with the lowest (2016-2018), intermediate (2002-2006) and abundant fishery (2011-2012). The X-axis indicates five ecoregions (OMAN-Oman Sea, NESA-North East Arabian sea, SEAS-South East Arabian sea, SBOB-South West Bay of Bengal, NBOB-Northwest Bay of Bengal) and Y-axis indicates Sea Surface Temperature (SST) in ^oC.

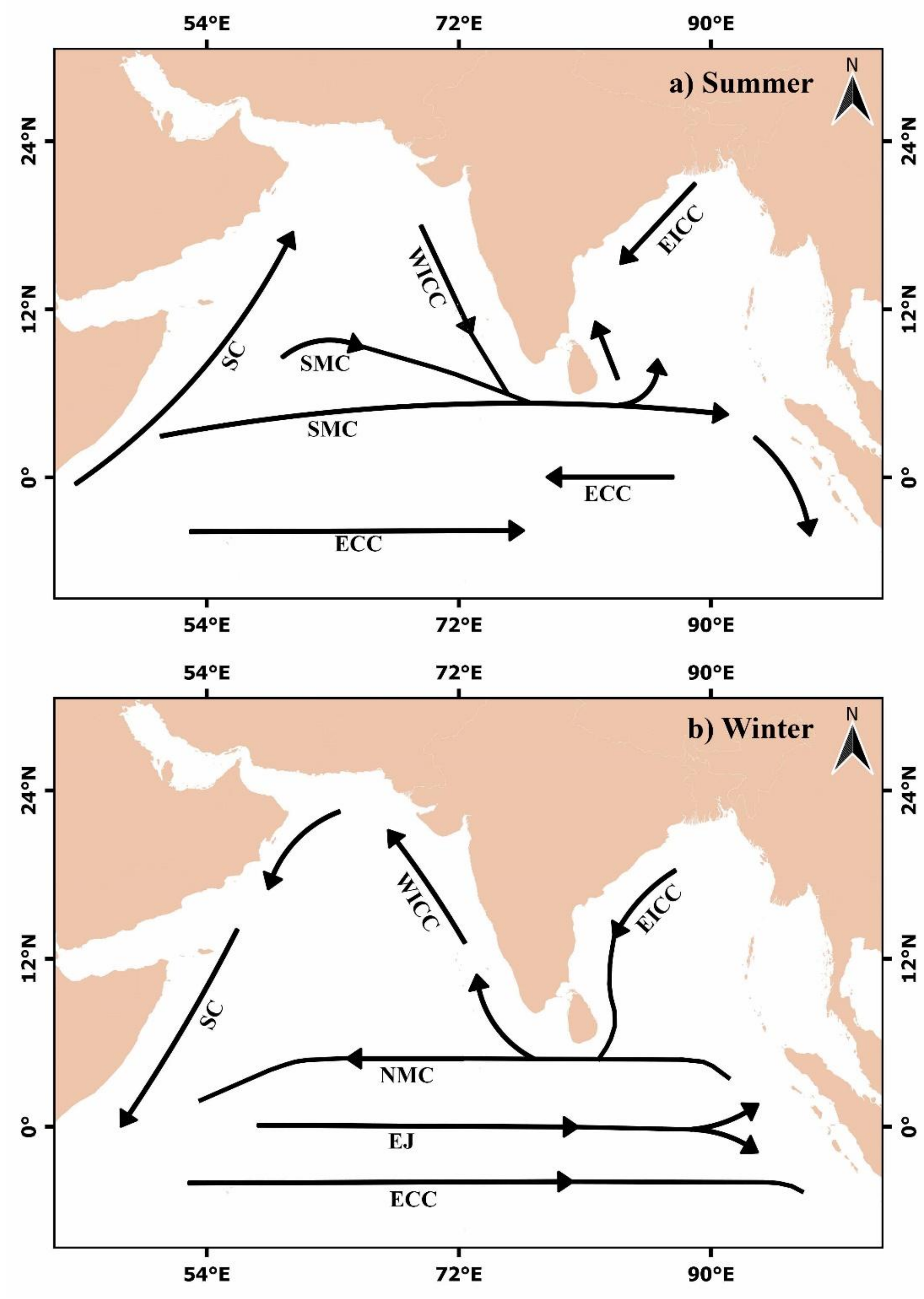

Figure S11 Schematic representation of major surface currents in the Indian Ocean during (a) the southwest monsoon (summer) and (b) the northeast monsoon (winter). The major currents are Northeast Monsoon Current (NMC), Equatorial Counter Current (ECC), Equatorial Jet (EJ), Somali Current (SC), Southwest Monsoon Current (SMC), West India Coastal Current (WICC) and East India Coastal Current (EICC). The EJ appears only during the transition period (summer to winter monsoon season) in April-May and November-December. The map was drawn using Adobe Photoshop CS6 (https://www.adobe.com/in/products/photoshop.html?promoid=PC1PQQ5T&mv=other).

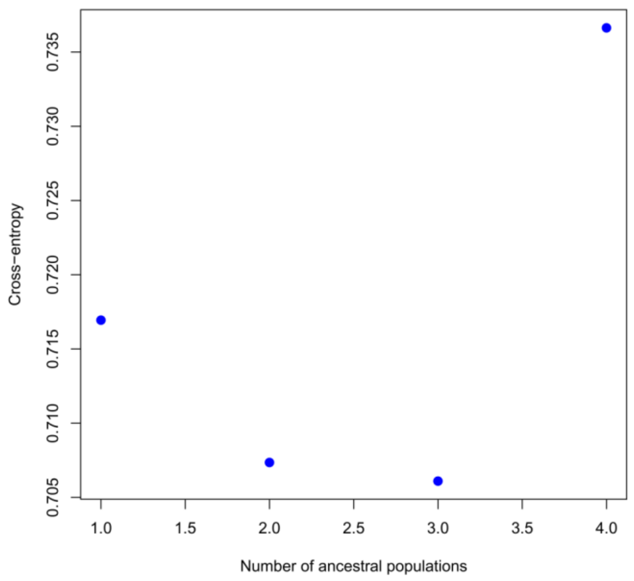

Figure S12 Least-squares estimates of ancestry proportions. The plot of the value of the cross-entropy criterion as a function of the number of populations in the R function 'snmf'.

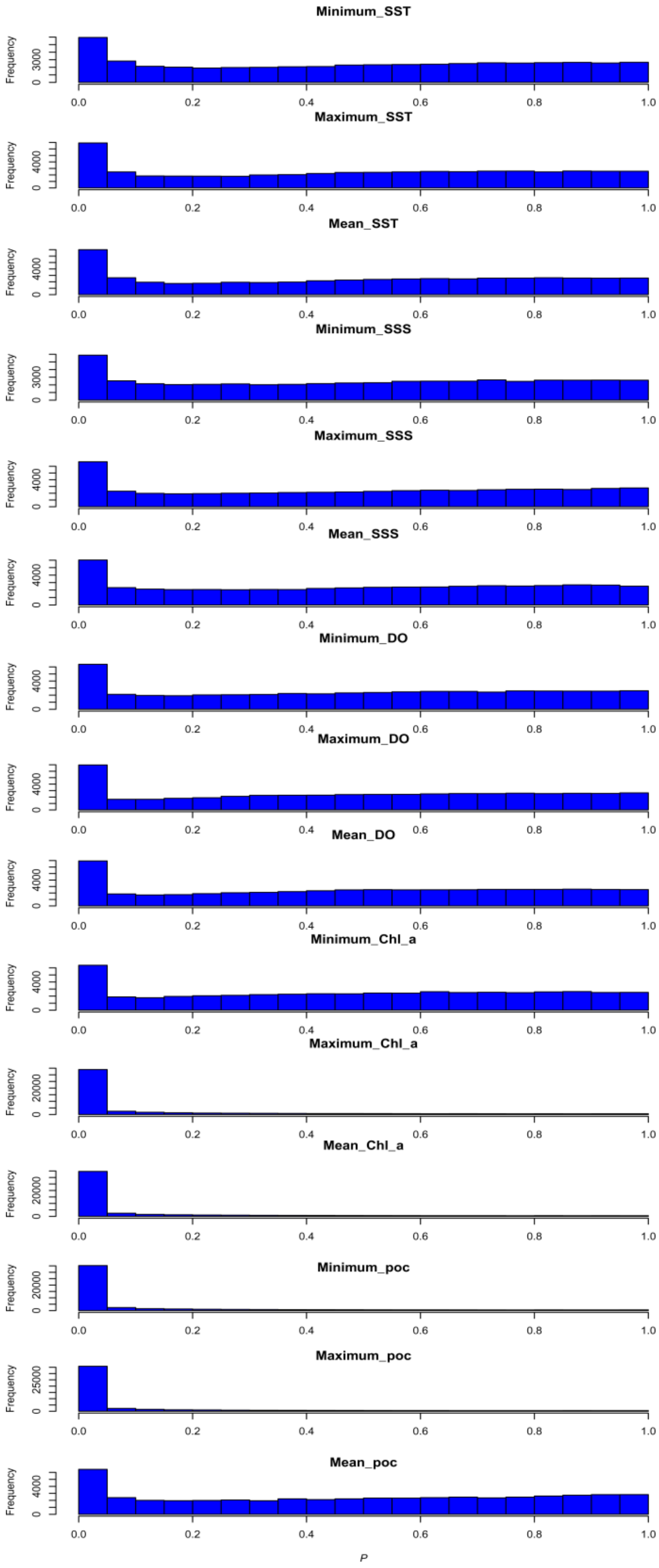

Figure S13 The p-values histogram from LFMM analysis. The plot was generated with LFMM in LEA (R package).

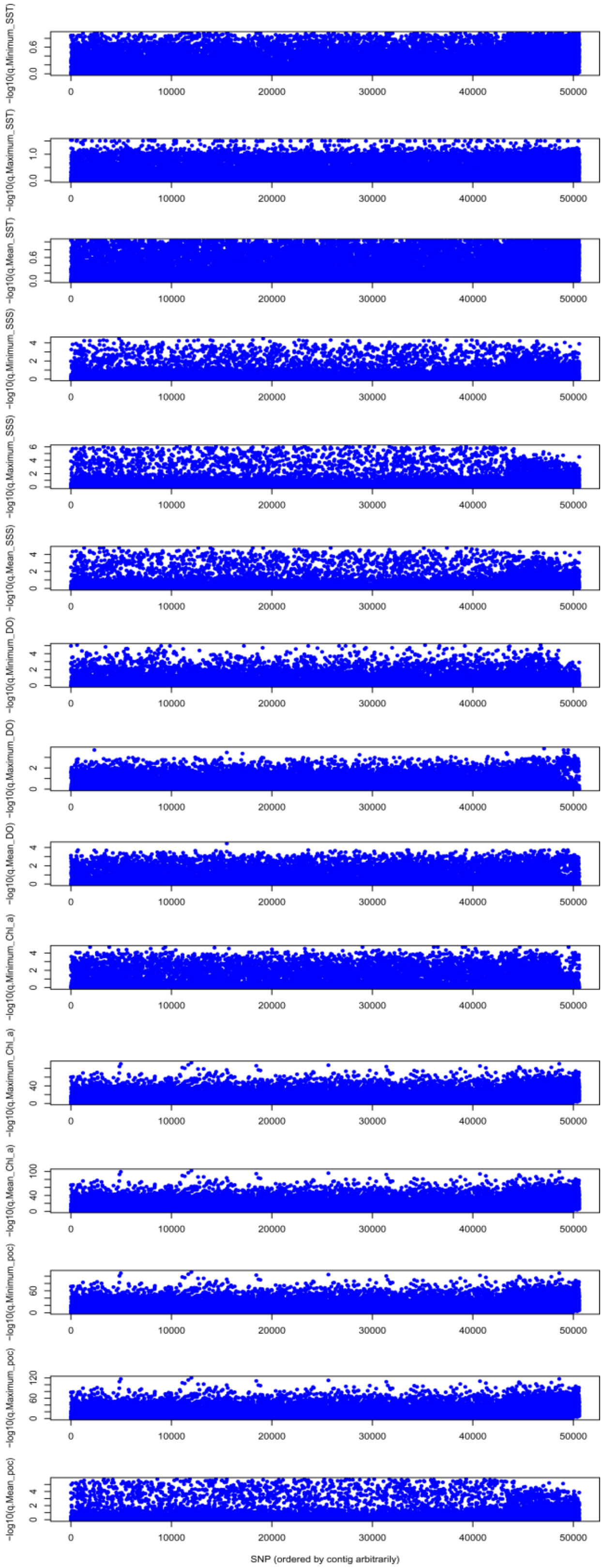

Figure S14 LFMM_Manhattan plot. The plot was generated with LFMM in LEA (R package).

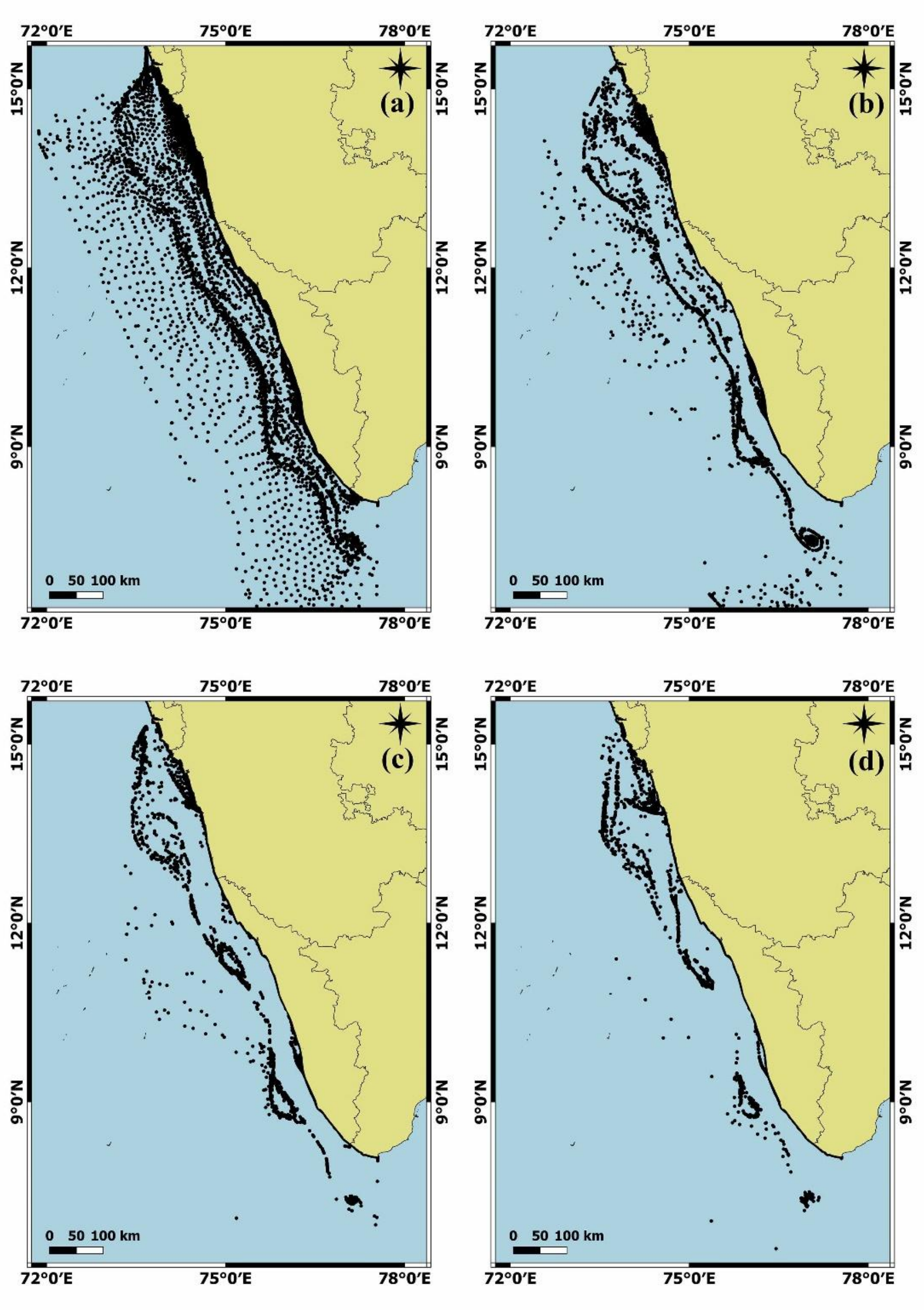

Figure S15 Transportation of particles from initial release during January 2014 after (a) 1 day (b) 5 days (c) 10 days (d) 20 days. The plot was generated with GMSH software (https://gmsh.info/).

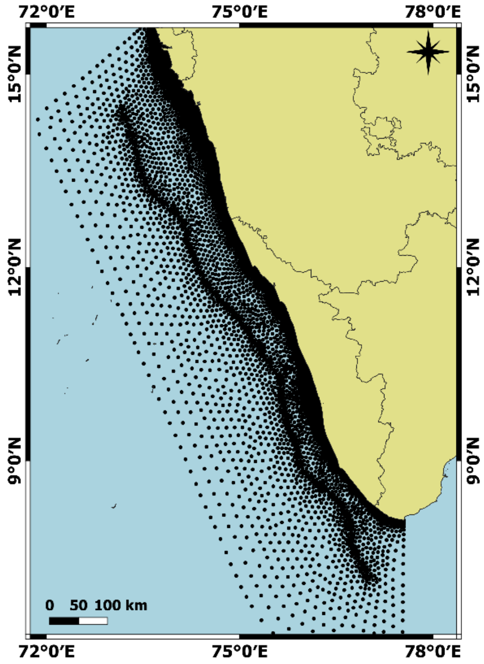

Figure S16 The initial release of 50000 particles at the surface layer of the southwest coast of India. The plot was generated with GMSH software (https://gmsh.info/).

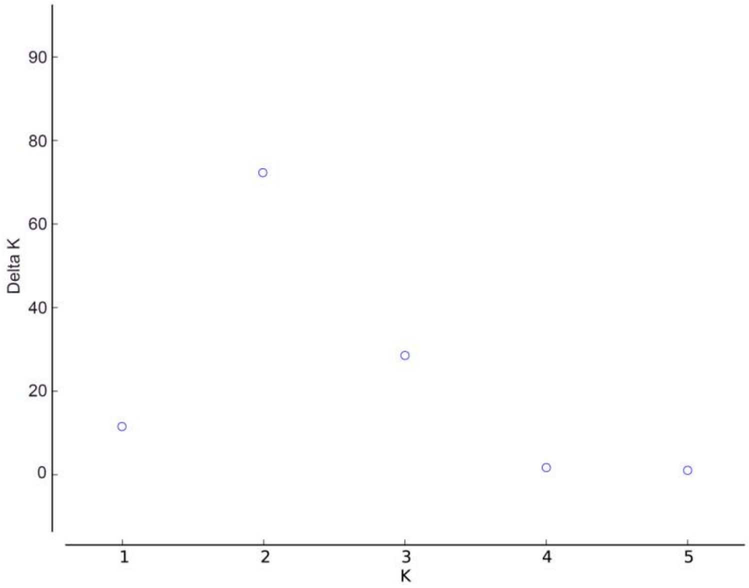

Figure S17 Delta *K* values for different numbers of assumed populations (*K*) in STRUCTURE analysis drawn by STRUCTURE HARVESTER.
